# Formin Activity and mDia1 Contribute to Maintain Axon Initial Segment Composition and Structure

**DOI:** 10.1007/s12035-021-02531-6

**Published:** 2021-08-30

**Authors:** Wei Zhang, María Ciorraga, Pablo Mendez, Diana Retana, Norah Boumedine-Guignon, Beatriz Achón, Michaël Russier, Dominique Debanne, Juan José Garrido

**Affiliations:** 1 Instituto Cajal, CSIC, 28002 Madrid, Spain; 2 Present Address: College of Chemistry and Molecular Engineering, Peking University, Beijing, China; 3 UNIS, INSERM, UMR 1072, Aix-Marseille Université, 13015 Marseille, France; 4 Alzheimer’s Disease and Other Degenerative Dementias, Centro de Investigación Biomédica en Red de Enfermedades Neurodegenerativas (CIBERNED), Madrid, Spain

**Keywords:** Axon initial segment, Formins, mDia1, Microtubules, AnkyrinG

## Abstract

The axon initial segment (AIS) is essential for maintaining neuronal polarity, modulating protein transport into the axon, and action potential generation. These functions are supported by a distinctive actin and microtubule cytoskeleton that controls axonal trafficking and maintains a high density of voltage-gated ion channels linked by scaffold proteins to the AIS cytoskeleton. However, our knowledge of the mechanisms and proteins involved in AIS cytoskeleton regulation to maintain or modulate AIS structure is limited. In this context, formins play a significant role in the modulation of actin and microtubules. We show that pharmacological inhibition of formins modifies AIS actin and microtubule characteristics in cultured hippocampal neurons, reducing F-actin density and decreasing microtubule acetylation. Moreover, formin inhibition diminishes sodium channels, ankyrinG and βIV-spectrin AIS density, and AIS length, in cultured neurons and brain slices, accompanied by decreased neuronal excitability. We show that genetic downregulation of the mDia1 formin by interference RNAs also decreases AIS protein density and shortens AIS length. The ankyrinG decrease and AIS shortening observed in pharmacologically inhibited neurons and neuron-expressing mDia1 shRNAs were impaired by HDAC6 downregulation or EB1-GFP expression, known to increase microtubule acetylation or stability. However, actin stabilization only partially prevented AIS shortening without affecting AIS protein density loss. These results suggest that mDia1 maintain AIS composition and length contributing to the stability of AIS microtubules.

**Supplementary Information:**

The online version contains supplementary material available at 10.1007/s12035-021-02531-6.

## Introduction

The axon initial segment (AIS) serves as a barrier that controls membrane protein diffusion and cytoplasmic transport towards the axon, maintaining neuronal polarity [1–5]. Simultaneously, the AIS is characterized by a high density of voltage-gated ion channels that allow action potential (AP) generation [6]. This high density of voltage-gated ion channels is achieved by a not completely understood protein complex formed by scaffold proteins, such as ankyrinG or βIV-spectrin, and a specialized actin and microtubule cytoskeleton [7]. The whole complex is characterized by a high stability and detergent extraction resistance [8], but has a surprising high degree of plasticity [9], which regulatory mechanisms at cytoskeleton level are relatively unknown. AnkyrinG is the main scaffold protein that anchors voltage-gated sodium channels [10, 11] and is bound to actin cytoskeleton through βIV-spectrin [12]. Moreover, ankyrinG can also bind to microtubules through EB1/3 proteins [13].

AIS actin and microtubules contribute to control axonal traffic and AIS structure [3, 5, 14–18]. However, our understanding of the mechanisms controlling AIS cytoskeleton dynamics is limited. AIS microtubules are highly fasciculated and contain post-translational modifications of tubulin, such as acetylation and detyrosination, which allow kinesin-1 to transport cargoes into the axon [19–21]. Changes in tubulin acetylation by the tubulin deacetylase HDAC6 contribute to modifications in AIS [21, 22]. A higher phosphorylation of the microtubule-associated protein Tau, due to V337M mutation, contributes to AIS shortening [23]. Recently, TRIM46 (tripartite motif–containing protein 46) was identified as a microtubule fasciculation protein at the AIS [15], and the microtubule-associated protein MAP7D2 was also localized to the proximal axon promoting kinesin-1-mediated cargo transport [17]. Besides that, AIS actin cytoskeleton is necessary to maintain the cytoplasmic barrier that impairs somatodendritic cargoes to enter the axon [5, 16, 24]. Different actin structures have been described in the AIS, such as actin patches, actin rings, or actin filaments [25]. The AIS also contains an actin-rich structure, the cisternal organelle (CO), supposed to be a calcium regulatory compartment [26] and related to AIS plasticity [27]. Some AIS actin-related proteins have been identified, such as synaptopodin, α-actinin-2, or myosin II and V [14, 24, 28]. Despite our increasing knowledge of actin- and microtubule-related proteins in the AIS, the mechanisms that regulate the crosstalk between both cytoskeleton components at the AIS are not fully understood.

Some studies have shown a mutual regulation of actin and microtubule stability in axons [29, 30]. The crosstalk between actin and microtubules has been well-studied during acquisition of neuronal cell shape or cell migration, modulated by shared regulators, such as MAPs, EBs, or formins [29]. Among them, EB1/EB3 and MAP7D2 are localized at the AIS [3, 17], while only one previous study points to localization of formin 2 at the proximal axon [31]. Fifteen different formins have been described in mammals [32], defined by their most conserved formin homology 2 (FH2) domain [33], which is the core element for formin physiological functions [34]. The regulatory mechanisms of formins are not fully understood, but their activation and autoinhibition depend on different mechanisms [35, 36]. Formins are a major class of effectors for cytoskeleton modulation, with the ability to modulate and coordinate actin and microtubule structures [34, 37, 38]. Among them, formins modulate actin filaments elongation and nucleation, and contribute to microtubule stabilization, promoting indirectly their acetylation [39]. Among their multiple physiological roles, formins are necessary to maintain apico-basal polarity in neuroepithelial and epithelial cells [40–42], and to regulate axonal actin structural dynamics [43]. Furthermore, formins are related to degenerative diseases and mental disorders [44].

Thus, we hypothesized that formins may play a role in the regulation of AIS structure. We found that formin inhibition modifies actin and microtubule AIS cytoskeleton and reduces scaffold and functional protein density at the AIS, both in cultured neurons and brain slices. This leads to a reduced intrinsic neuronal excitability. Moreover, formin activity has a dual role in microtubule or actin filament regulation leading to the maintenance of AIS length or AIS protein density. Finally, we have identified mDia1 formin as a main formin to maintain AIS protein density and AIS length.

## Materials and Methods

### Animals

Animals, Swiss CD1 mice, were housed in the Experimental Animal facility at Cajal Institute and maintained according to national legislation (53/2013, BOE no. 1337) and guidelines of the Council of the European Communities (2010/63/UE). Protocols were previously approved by the CSIC bioethics committee.

### Reagents and Plasmids

The following reagents were used in the study: SMIFH2 (S4826), Tubastatin A hydrochloride (SML0044), and CK-666 (SML-0006) were purchased from Sigma-Aldrich, and jasplakinolide (2792) from TOCRIS. The interference RNA plasmids of mouse for mDia1 were obtained from Origene with the RFP tag. Two effective targets were identified: 5′-TGCCACTGACGAGAAGGACAAGTTTGTTG-3′ (Cat# TF500527D, shmDia1-1) and 5′-GAAGGAATCCTACTGCTGGTCAGAGCCAT-3′ (Cat# TF500527A, shmDia1-2). HDAC6 interference RNA plasmids with GFP tag from Origene have been previously described [21]. AnkG-GFP (270 kD) plasmid was a kind gift from Dr Van Bennett laboratory. pEGFP-N1, as a control of recombinant DNA over-expression, was bought from Clontech (Cat# 6085-1). The GFP-CA-mDia1 vector expressing a constitutively active form of mDia1 (plasmid # 45583) and the recombinant vector of EB1-GFP (plasmid # 39299) were obtained from Addgene.

### Cell Cultures

Mouse hippocampal neurons were prepared as previously described [45, 46]. Briefly, mice hippocampi were dissected, incubated in a 0.25% trypsin solution in Ca^2+^/Mg^2+^ free Hank’s buffered salt solution (HBSS) and dissociated using fire-polished Pasteur pipettes. Neurons were placed on poly-lysine-coated coverslips (1 mg/ml) at a density of 6000 cells/cm^2^ for 2 h in a plating medium (MEM, 10% horse serum, 0.6% glucose and Glutamax-I). Then coverslips were inverted and transferred to culture dishes containing an astrocyte monolayer in a neuronal medium (Neurobasal, B27 supplement and Glutamax-I). A 5 μM 1-β-d-arabinofuranosylcytosine (AraC) was added 2 days after to curb glial cells proliferation. Neurons were maintained replacing one-third of neuronal medium every week. In the case of pharmacological treatments in the absence of glial cell layer, coverslips were transferred to plates containing glial cell–conditioned medium.

Nucleofection was performed after neuronal dissociation using the P3 Primary Cell 4D-Nucleofector^TM^ X Kit L for primary mammalian neural cells (Cat# V4XP-3012, Lonza), using the CL133 program, 2 × 10^6^ cells and 3 μg of total DNA for each nucleofection. After nucleofection, neurons were plated at a density of 15.000 cells/cm^2^.

Neuronal transfection was performed after plating at the times described in the “Results” section. Hippocampal neurons (20,000 cells/cm^2^) were transfected with Lipofectamine 2000 (Cat#11668-030, Invitrogen) and maintained for 24 h or 48 h.

Neuro-2a cells were cultured in DMEM containing 10% FBS and glutamine (2 mM). Cells were transfected using Lipofectamine 2000 (Invitrogen) following manufacturer instructions, and kept for 3 days before fixation and immunocytochemistry.

### Brain Slice Cultures and Electrophysiology

Slice cultures containing the hippocampus and entorhinal cortex were obtained from postnatal day 5–7 rats as previously reported [47]. Slices (350 μm) were cut in ice-cold sucrose-based slicing solution (280 mM sucrose, 26 mM NaHCO3, 1.3 mM KCl, 1 mM CaCl2, 10 mM MgCl2, 11 mM d-glucose, and 2 mM kynurenate) oxygenated with 95% O2/5% CO_2_ and were collected in the slicing solution added with 5% horse serum and 0.6% HEPES (1 M), and maintained for 1 h at room temperature in oxygenated (95% O2/5% CO2) standard artificial cerebrospinal fluid (125 mM NaCl, 2.5 mM KCl; 0.8 mM NaH2PO4, 3 mM NaHCO3, 3 mM CaCl2, 2 mM MgCl2, and 11 mM d-glucose). Each slice was placed on 320-mm latex PTFE hydrophilic membranes (Merck-MilliporeMillicell) inserted into 35-mm Petri dishes containing 1 ml of culture medium (25 ml MEM, 13.5 ml HBSS, 12.5 ml horse serum, 0.5 ml penicillin/streptomycin, 0.8 ml glucose solution (1 M), 0.1 ml ascorbic acid solution (1 mg/ml), 0.4 ml 4-(2-hydroxyethyl)-1-piperazine ethanesulfonic acid (HEPES) (1 M), 0.5 ml B27, and 8.95 ml water) and kept at 34 °C, 95% O2–5% CO_2_. To arrest glial proliferation, 5 μM Ara-C was added to the culture medium and removed the day after. Cultures at 7 DIV were treated in the culture medium for 3 h before recording with 30 μM SMIFH2 diluted in DMSO or with DMSO alone (1%).

Whole-cell patch-clamp recordings were obtained from CA3 pyramidal neurons. The external solution contained (mM): 125 NaCl, 26 NaHCO3, 3 CaCl2, 2.5 KCl, 2 MgCl2, 0.8 NaH2PO4, and 10 d-glucose, and was equilibrated with 95% O2-5% CO_2_. Patch-pipettes (5–10 MΩ) were filled with a solution containing (mM) 120 potassium gluconate, 20 KCl, 0.5 EGTA, 10 HEPES, 2 Na2ATP, 0.3 NaGTP, and 2 MgCl2, pH 7.4. Recordings were made at 30 °C in a temperature-controlled recording chamber (Luigs & Neumann). The voltage and current signals were acquired on a PC computer fed by a Digidata1440A at a sampling frequency of 10 kHz for input-output curves and 40 kHz for spike threshold measurements. CA3 neurons were identified by their firing behavior. Neurons were held at their resting membrane potential (~−77 mV). Input-output curves were established by 10 pA increments of current step, and spikes were counted. Rheobase and gain were measured for each neuron as previously reported [48]. Spike threshold was measured as the first value of voltage corresponding to a rate exceeding 30 mV/ms.

Recordings in P26 mice acute slices were from layer 2/3 neurons visually identified using infrared video microscopy. Slices were perfused with oxygenated artificial cerebrospinal fluid containing the following: 126 mM NaCl, 26 mM NaHCO3, 2.5 mM KCl, 1.25 mM NaH2PO4, 2 mM MgSO4, 2 mM CaCl2, and 10 mM glucose (pH 7.4). Recording were performed in the presence of kynurenic acid (2 mM) and SR95531 (Gabazine, 10 μM) to block glutamate and GABAA receptor–mediated synaptic currents. Patch-clamp electrodes contained intracellular solution composed of 70 mM K^+^-gluconate, 70 mM KCl, 2 mM NaCl, 2 mM MgCl2, 10 mM HEPES, 1 mM EGTA, 2 mM MgATP, and 0.3 mM Na2GTP (pH 7.3). A 3-s ramp between 0 and 250 pA was used to calculate threshold for action potential generation.

### Immunocytochemistry

Neurons or Neuro2a cells were fixed in 4% paraformaldehyde (PFA) for 15 min, washed in phosphate buffered saline (PBS), treated with 50 mM NH_4_Cl for 10 min, and incubated at RT in blocking buffer for 1 h (0.22% gelatin, 0.1% Triton X-100 in PBS). Primary antibodies were incubated for 1 h diluted in blocking buffer. The primary antibodies used were rabbit polyclonal anti-DIAPH1 (1:70, Cat# PA5-27607, Thermo Fisher Scientific), knockout-validated rabbit polyclonal anti-DAPH1 (1:100, ab129167, Abcam), mouse monoclonal anti-mDia1 (1:50, 610849, BD, previously used in [49]), mouse monoclonal anti-AnkG (IgG2a, 1:150, clone N106/36, NeuroMab), mouse monoclonal anti-PanNaCh (IgG1, 1:100, Cat# S8809, Sigma-Aldrich), rabbit polyclonal anti-pMLC (1:200, Cat# PA5-17727, Thermo Fisher Scientific), rabbit polyclonal anti-βIV-Spectrin (1:1000, a gift of Dr Matthew Rasband, Baylor College of Medicine), chicken polyclonal anti-MAP2 (1:5000, Cat# ab5392, Abcam), mouse monoclonal anti-acetylated-tubulin (IgG2b, 1:5000, Cat# T7451, Sigma-Aldrich), mouse monoclonal anti-α-tubulin (IgG1, 1:5000, Cat# T6199, Sigma-Aldrich), rabbit polyclonal anti-KIF5C (1:200, Cat# ab5630, Abcam), and rabbit polyclonal anti-Synaptopodin (1:500, Cat# S9442, Sigma-Aldrich). Coverslips were incubated with Alexa coupled isoform specific secondary antibodies (1:1000) in blocking buffer for 45 min. F-Actin was stained using Alexa Fluor-568-conjugated Phalloidin (1:100, Cat# A12380, Thermo Fisher Scientific). Coverslips were mounted with Fluoromount-G (Southern-Biotech). Images were acquired on a Leica SP5 confocal microscope at a resolution of 1024 × 1024 pixels.

### Immunohistochemistry

Brains from mice P26-P30 days old were quickly removed and acute coronal slices (300 μm) containing sensorimotor cortex were cut with a vibratome (4 °C) in a solution containing: 234 mM sucrose, 11 mM glucose, 26 mM NaHCO_3_, 2.5 mM KCl, 1.25 mM NaH_2_PO_4_, 10 mM MgSO_4_, and 0.5 mM CaCl_2_ (equilibrated with 95% O_2_–5% CO_2_). SMIFH2 treatment was carried out at a concentration of 15 or 30 μM for 3 h, and the same volume of vehicle DMSO was used as control. Slices were fixed 30 min at RT with 4% PFA and washed in PBS. Slices were incubated for 2 h at RT with PBS containing 0.1% TritonX-100 and 10% goat serum, followed by 4 °C overnight incubation with anti-AnkG antibody (IgG2a, 1:150) in PBS containing 0.1% TritonX-100 and 1% goat serum. After secondary antibody incubation (1:500) for 2 h at RT, sections were counterstained with bis-benzimide (5 μg/ml, Cat# B2261, Sigma-Aldrich) for nuclei staining and mounted in Fluoromount-G. Images were acquired on a Leica SP5 confocal microscope using Leica DM6000B 40× 1.25 N.A. oil objective. Sections were imaged in z stacks with a 0.5-μm step size. For qualitative analysis, a Z-projection was obtained in the Fiji-ImageJ software. We measured individual AISs in cortical layers II/III, IV, and V/VI. Cortical layers were distinguished according to nuclei patterns. In the case of CA1 staining quantification, total ankyrinG fluorescence was measured in the whole CA1 region as shown in figures.

### Fluorescence Recovery After Photobleaching

For FRAP experiments, hippocampal neurons were plated directly on 35-mm culture dishes with glass bottom (Ibidi). Neurons were plated at a density of 20.000 cells/cm^2^ and supporting glial cells were place on inverted 24-mm coverslips. Neurons were lipofected with AnkyrinG-GFP at 8 DIV and kept for 24 h. Prior to FRAP experiment, neurons were treated with 15 μM SMIFH2 or DMSO for 3 h. Live imaging was performed on a Leica SP5 inverted confocal microscope, using 63× 1.40 NA oil objective and maintaining 37 °C and 5% CO_2_ in the live cell chamber. Time-lapse images were acquired using bidirectional scan at 1400 Hz with the format of 256 × 256 pixels and the pinhole size was 2 airy units. AIS was distinguished by AnkyrinG-GFP concentration at the proximal axon. A ROI with an area of 5 μm × 5 μm on the proximal axon was photobleached at 100% 488-nm Laser Line power with 25% Argon Laser Power, reducing local fluorescence by 80–95%. FRAP was performed with 10 frames of 0.1-s intervals for pre-bleach and 15 s for bleach with 0.1-s intervals, followed by post-bleach with 240 frames of 0.5-s intervals. FRAP analysis was performed as previously described [13]. The recovery curve was generated by pooling together at least 15 neurons in each condition and averaging each frame the recovery rate. The maximum recovery percentage was obtained through averaging recovery data in 20 s at the plateau from every neuron in Sigmaplot v12.5 (Systat Software Inc., San Jose, CA, USA).

### AIS Parameter Quantification

Confocal microscope settings were adjusted to prevent signal saturation and the images were taken in z stacks with a 0.5-μm step size. Images were obtained randomly. All images from every experiment were obtained using the same settings, standardized in the control samples of each experiment. For quantitative analysis, all coverslips in each experiment experienced the same procedures for labeling. AIS was identified by ankyrinG staining. A stack of 1.5-μm 3 confocal sections containing the AIS was used to quantify AIS parameters. Analysis was performed using the Fiji-ImageJ software (NIH) drawing a line along the axon, starting at the soma. Data were smoothed every 1 μm using the Sigma Plot 12.5 software. AIS start, maximum, and end positions were determined as described previously [50]. Total fluorescence intensity for each marker was obtained by adding fluorescence intensity values from start to end position after determining AIS length using ankyrinG staining. Data where normalized in each experiment to the value of the mean fluorescence in control neurons. For AIS profiles data were normalized to the maximum fluorescence value in control neurons.

### Statistical Analysis

All statistical analyses were carried out in GraphPad Prism 5 and Sigmaplot v12.5. Sample Gaussian distributions were first assessed for normality using the D’Agostino and Pearson omnibus test or Shapiro-Wilk’s test. Statistical analysis was performed using a two tailed t-test for two group comparisons: Unpaired *t*-test for parametric data and a post hoc Mann-Whitney test for non-parametric data. For multiple group comparisons, one-way analysis of variance with Tukey post hoc test was used for parametric data and a post hoc Kruskal-Wallis with Dunn’s test for non-parametric data. Graphs were represented as the mean ± SEM. Differences were considered significant when *p*-value less than 0.05, and represented as *p < 0.05, **p < 0.01, *** < 0.001.

## Results

### Formin Inhibition Modify AIS Protein Density

**Fig. 1:**
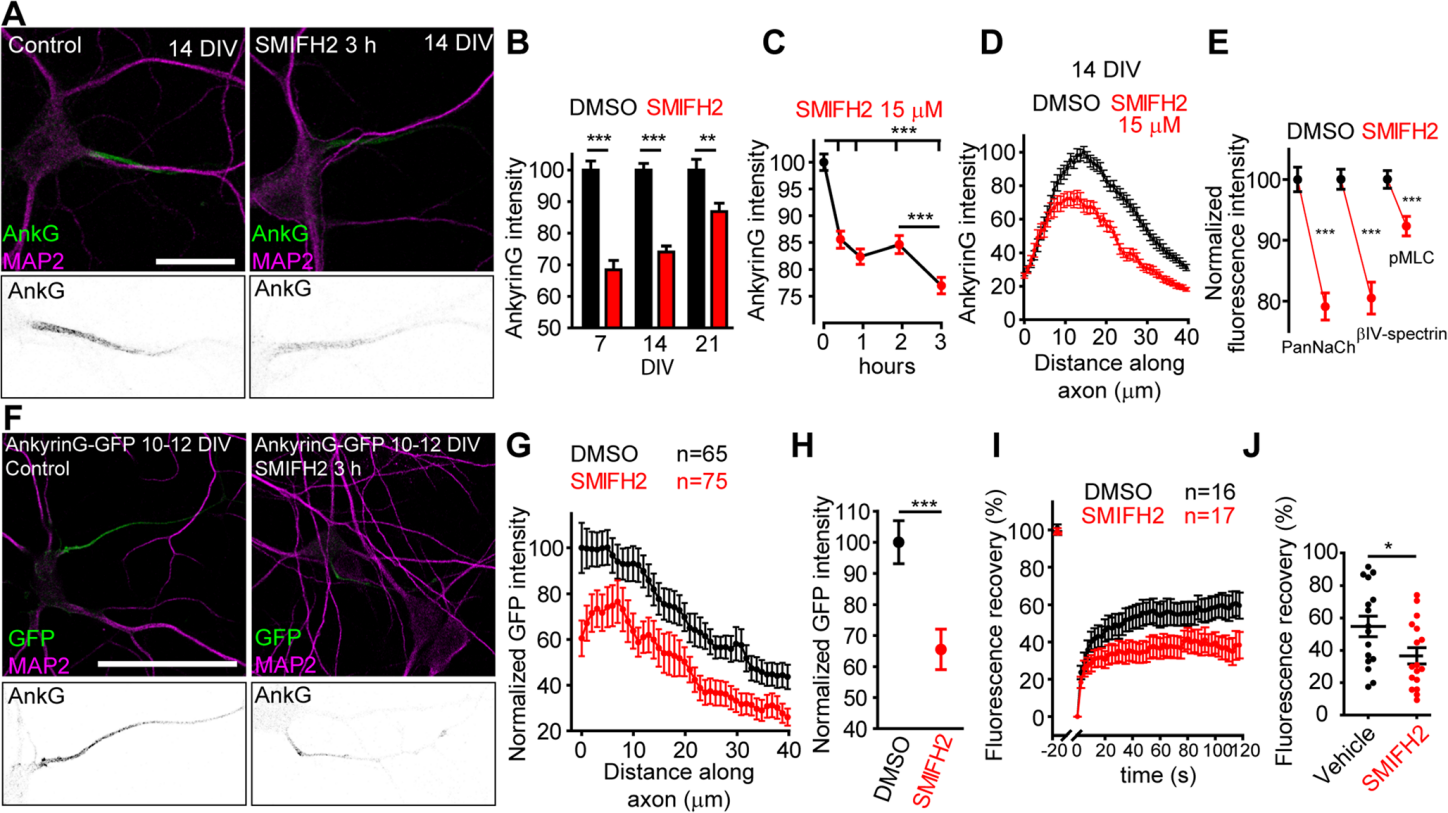
Formin inhibition decreases AIS protein density in hippocampal neurons. **A** A total of 14 DIV hippocampal neurons treated for 3 h with DMSO or 15 μM SMIFH2. AISs and somatodendritic compartment are identified by ankyrinG (green) and MAP2 (magenta). Bottom panels show AIS magnifications. Scale bar = 20 μm. **B** Normalized ankyrinG intensity in 7, 14, and 21 DIV hippocampal neurons treated for 3 h with vehicle (black bars) or 15 μM SMIFH2 (red bars). **p < 0.01, ***p < 0.0001, Mann-Whitney test (*n* = 150/experimental condition). **C** Normalized ankyrinG fluorescence intensity in 14 DIV neurons treated with DMSO (black dots) or 15 μM SMIFH2 (red dots) for 0.5, 1, 2, and 3 h. ***p < 0.001, Kruskal-Wallis, Dunn’s multiple comparison test (*n* = 150/experimental condition). **D** AnkyrinG intensity profile in 14 DIV neurons along the AIS. **E** Normalized AIS sodium channels (PanNaCh), βIV-spectrin, and phospho-myosin light chain (pMLC) fluorescence intensity in 14 DIV neurons treated with DMSO (black) or 15 mM SMIFH2 (red) for 3 h. ***p < 0.001, unpaired *t*-test (*n* = 120). **F** 10 DIV hippocampal neurons transfected with AnkyrinG-GFP for 48 h and exposed to DMSO or 15 μM SMIFH2 for 3 h. Bottom panels show AIS magnifications. Scale bar = 50 μm. **G**, **H** Normalized AIS GFP fluorescence intensity in vehicle (black) or SMIFH2 (red)-treated neurons (**H**) and GFP signal profile along the AIS (**G**). ***p < 0.001, unpaired *t* test. **I** Percentage of AIS AnkyrinG-GFP fluorescence recovery for 120 s after photobleaching in FRAP experiments. **J** Mean maximal GFP recovery. *p < 0.05, unpaired *t* test. Graphs show the mean ± SEM of the indicated number of data obtained from at least three independent experiments.

To determine the effects of formin activity on AIS structure and protein composition, we treated neurons with SMIFH2, an inhibitor of formin conserved FH2 domain [34, 37, 51]. Treatment with SMIFH2 (15 μM) in 14 DIV-cultured hippocampal neurons led to a significant ankyrinG decrease after 3 h of application (−25%, Fig. 1A, B) that was detected after 30 min (−15%, Fig. 1C). This decrease was more pronounced in young neurons (7 DIV, −32%), than in older neurons (21 DIV, −15%) after 3 h of application (Fig. 1B), and was due to a decrease all along the AIS (Fig. 1D). Moreover, a similar reduction was also detected in 14 DIV neurons for other AIS proteins (Fig. 1E), such as βIV-spectrin (−20%), voltage-gated sodium channels (PanNaCh, −20%), and phospho-myosin light chain (pMLC, −10%), suggesting an effect on the underlying AIS cytoskeleton (Fig. S1). To discard that SMIFH2 treatment was generating an indirect effect on neurons through formin inhibition in astrocytes, we applied SMIFH2 to neurons in the absence of astrocytes (Fig. S2A) and detected again an ankyrinG reduction (−28.80 ± 1.89%) compared to DMSO-treated neurons. Moreover, after additional incubation in new plates containing astrocyte-conditioned neuronal medium for 6 h (Fig. S2B), a further decrease of 10% of ankyrinG intensity was observed. After 24 h, ankyrinG decrease showed an inverse correlation with increased expression of the somatodendritic marker MAP2 along the AIS, entering the axon (Fig. S2C–E). Finally, we analyzed whether ankyrinG decrease could be due to calpain activation as previously described [45, 52]. Hippocampal neurons (14 DIV) were pre-treated with the calpain inhibitor MDL-28170 (50 nM) for 1 h, and then neurons were incubated with SMIFH2 (15 μM) or DMSO for 3 h (Fig. S3). Our results show that ankyrinG decrease is not impaired by calpain inhibition, and other mechanisms are responsible for AIS protein reduction.

In order to confirm these results, 10 DIV hippocampal neurons were transfected with a plasmid expressing ankyrinG-GFP for 48 h and treated with SMIFH2 for 3 h (Fig. 1F). Analysis of the GFP signal at the AIS confirmed the results obtained for endogenous ankyrinG. The ankyrinG-GFP signal was reduced by a 35% along the AIS (Fig. 1G, H). In addition, ankyrinG-GFP signal was analyzed “in vivo” in hippocampal neurons after SMIFH2 treatment (Fig. 1I, J). Fluorescence recovery after photobleaching (FRAP) experiments demonstrated that ankyrinG-GFP signal recovery after photobleaching was significantly lower after SMIFH2 treatment (36 ± 5% vs 55 ± 6% in control neurons, Fig. 1J).

**Fig. 2:**
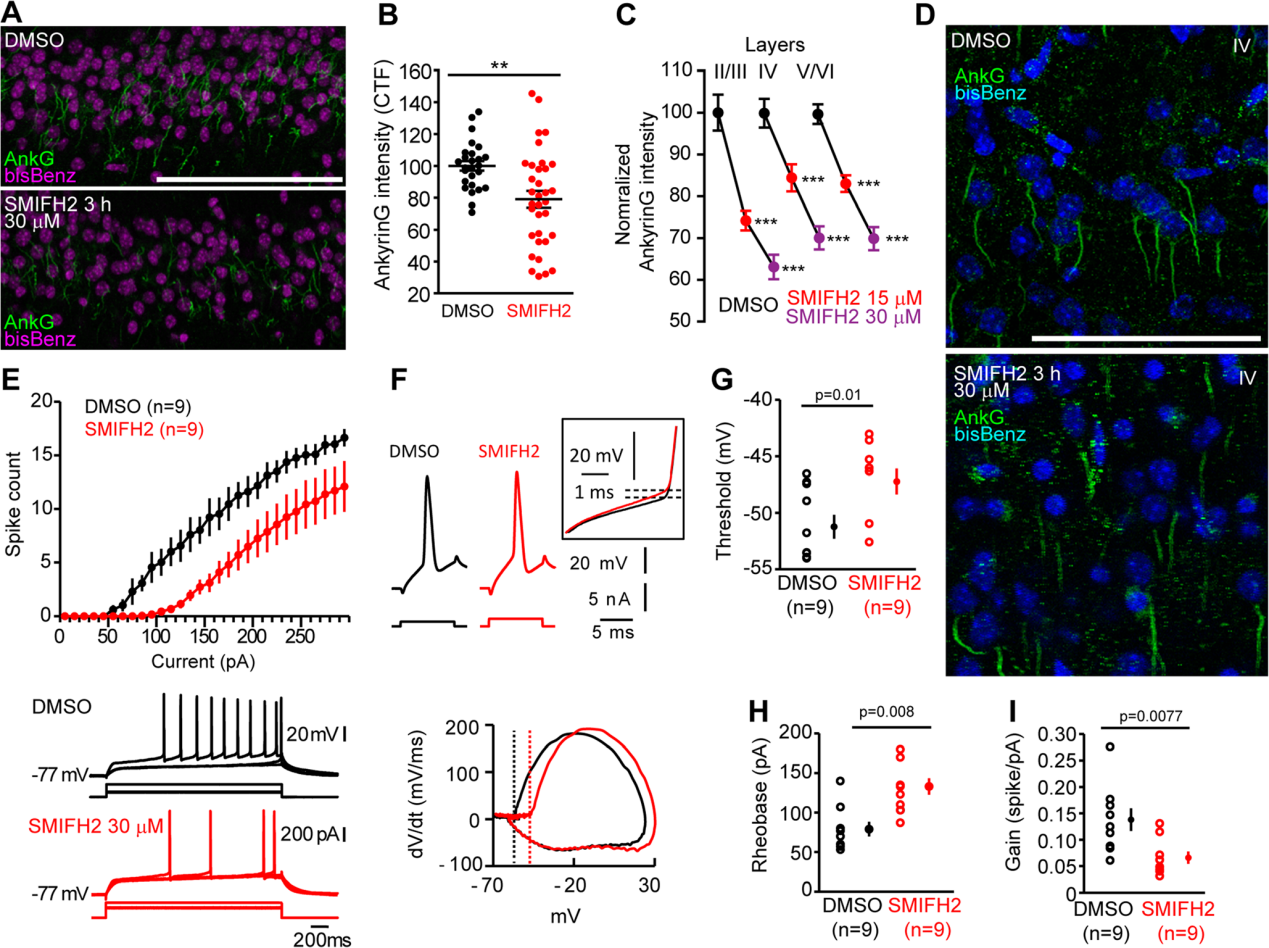
Formin inhibition decreases ankyrinG intensity in brain slices and reduces neuronal intrinsic excitability. **A** Brain slices (300 μm) containing hippocampi from P26 mice were treated with DMSO or 30 μM SMIFH2 for 3 h. Brain slices were stained to visualize ankyrinG (green) and nuclei (magenta). Scale bar = 100 μm. **B** Quantification of corrected total fluorescence intensity (CTF) in CA1 regions. **p < 0.01, unpaired *t* test. Each dot represents a CA1 zone as shown in **A**. Analysis was done in 4 independent experiments using 8 animals, and a total of 26 (control) and 34 (SMIFH2) slices. **C** Normalized ankyrinG intensity in DMSO (black) or 15 μM (red) and 30 μM SMIFH2 (magenta) in AISs of layers II/III, layer IV, and layers V/VI of P26 mice coronal sensorimotor cortex slices (300 μm). ***p < 0.001, Kruskal-Wallis, Dunn’s multiple comparison test were analyzed. Data are represented as the mean ± SEM and acquired in 150 AISs, for each condition and layer, from three independent experiments. **D** Representative images of ankyrinG-stained cortical sections treated with DMSO or SMIFH2. **E** CA3 neurons from rat hippocampal slice organotypic cultures treated for 3 h in the presence of 30 μM SMIFH2 (red) or DMSO alone (control, black) were recorded under current-clamp and the number of evoked action potentials plotted against injected current. Representative traces are shown in the bottom panel. **F**, **G** Spike threshold, dV/dt curves, and representative traces (inset = focus on spike threshold) in control and SMIFH2-treated slices. **H**, **I** Rheobase (**H**) and gain (**I**) in control (black) and SMIFH2 (red)–treated slices

We next analyzed whether this ankyrinG decrease by formin inhibition also happens in P26 mice brain slices (300 μm) after 3-h incubation with vehicle (DMSO) or SMIFH2 (15 and 30 μM). We measured ankyrinG fluorescence intensity in hippocampus (Fig. 2A) and cortical layers II/III, IV, or V/VI (Fig. 2D). AnkyrinG intensity was reduced around 20% after 30 μM SMIFH2 treatment (79 ± 5% vs 100 ± 3% in control slices) in CA1 hippocampal areas (Fig. 2B). SMIFH2 (15 μM or 30 μM) decreased ankyrinG intensity in an analogous percentage (−20% or −30%, respectively) in individual AISs in all cortical layers (Fig. 2C, D), showing a SMIFH2 concentration–dependent ankyrinG reduction (Fig. 2C). These data suggest that formin activity is necessary to maintain AIS protein density, both in cultured neurons and in brain slices.

### Formin Inhibition Decreases Neuronal Intrinsic Excitability

Since formin inhibition affects AIS structure, we analyzed whether action potential generation and neuronal excitability was altered. We treated organotypic slice cultures with SMIFH2 or DMSO for 3 h and recorded in CA3 pyramidal neurons. SMIFH2 treatment produced a marked reduction in intrinsic excitability (Fig. 2E). This reduction in excitability was associated with a higher rheobase (from 79 ± 9 pA, *n* = 9 to 133 ± 10 pA, *n* = 9, Mann-Whitney, *p* < 0.01; Fig. 2H), and a reduction in the gain (from 0.14 ± 0.02 to 0.07 ± 0.01 spike/pA, Mann-Whitney, *p* < 0.01; Fig. 2I). However, membrane capacitance and input resistance were found to be unchanged (DMSO: 293 ± 5 pF and 279 ± 7 MΩ, *n* = 9, SMIFH2: 313 ± 8 pF and 226 ± 5 MΩ, Mann-Whitney *p* > 0.1 and *p* > 0.05 respectively). Furthermore, the voltage threshold of the action potential was found to be depolarized after treatment with SMIFH2 (DMSO: −51 ± 1 mV, *n* = 9, SMIFH2: −46.9 ± 1.1 mV, *n* = 9; Mann-Whitney, *p* = 0.01; Fig. 2F, G). A similar depolarization of the spike threshold was found in P26 cortical neurons in acute brain slices (DMSO: −39.04 ± 1.06 mV, *n* = 12, SMIFH2: −36.08 ± 0.71 mV, *n* = 13; Mann-Whitney, *p* = 0.009). No significant change was observed in spike amplitude in hippocampal neurons (DMSO: 69.5 ± 3.2 mV, *n* = 9 vs SMIFH2: 66.6 ± 2.4 mV, *n* = 9, Mann-Whitney *U*-test, *p* = 0.66). These results suggest that formin inhibition has an impact on action potential generation due to ankyrinG and sodium channel reduction at the AIS.

### Formin Inhibition Decreases AIS F-Actin and Microtubule Acetylation

**Fig. 3:**
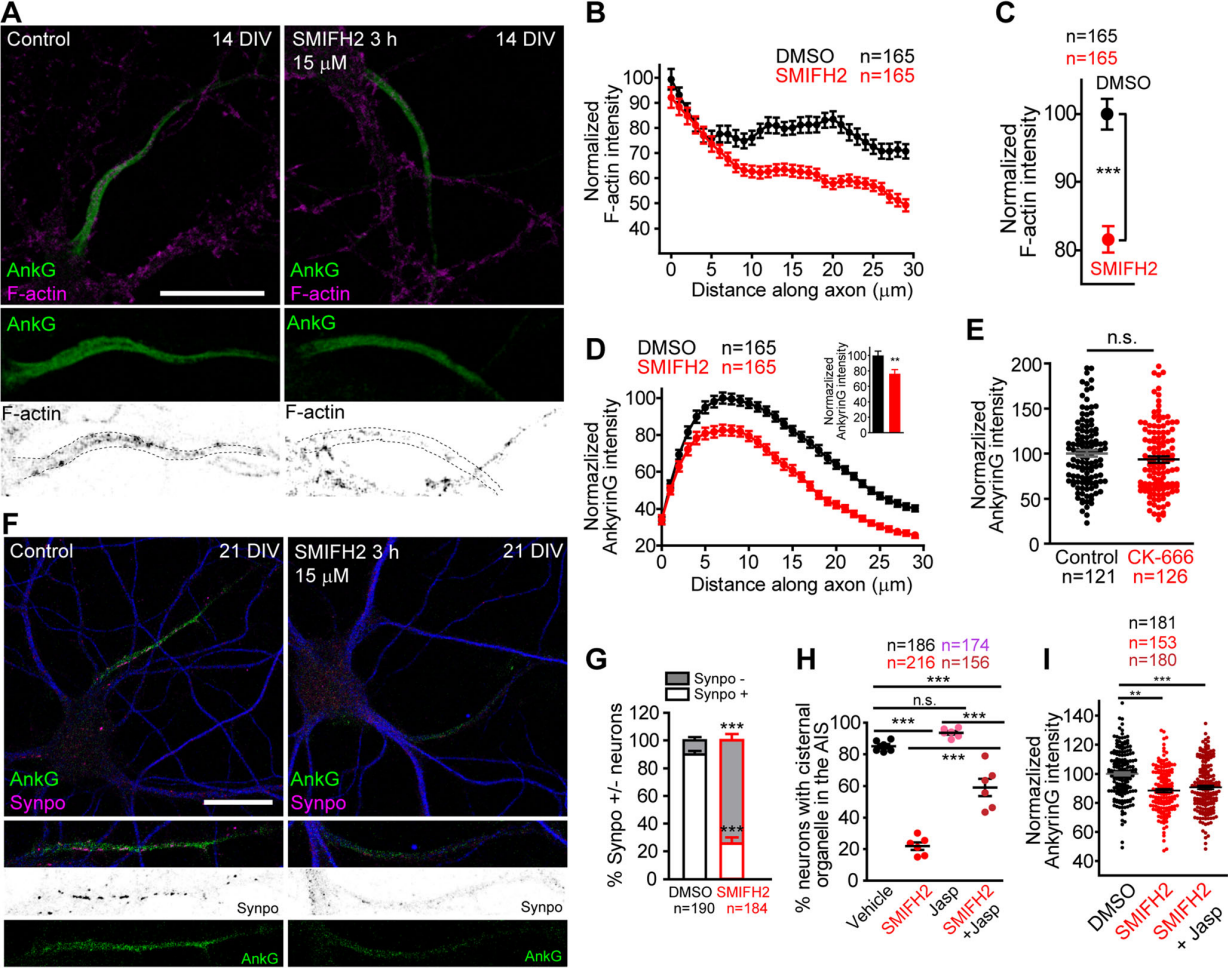
Formin inhibition decreases F-actin intensity in the AIS and affects cisternal organelle. **A** A total of 14 DIV neurons treated with DMSO or 15 μM SMIFH2 for 3 h. AnkyrinG staining (green) identifies the AIS and Alexa Fluor 568 Phalloidin (magenta) stains F-actin. Magnifications of AISs are shown in bottom panels. Scale bar = 20 μm. **B**, **C** Normalized F-actin intensity along the AIS (**B**) and normalized total F-actin within the AIS (**C**) in vehicle (black) or SMIFH2 treated neurons (red). ***p < 0.0001, Mann-Whitney test. **D** Normalized ankyrinG profile and total ankyrinG intensity within the AIS in the same neurons quantified in **B** and **C**. **E** Normalized ankyrinG fluorescence intensity in 14 DIV hippocampal neurons treated with the Arp2/3 inhibitor CK-666 (50 μM). **F** AnkyrinG (green) and cisternal organelle (synaptopodin, magenta) staining in 21 DIV hippocampal neurons treated with vehicle DMSO or 15 μM SMIFH2 for 3 h. MAP2 staining (blue) identifies dendrites. Magnifications of AIS region are shown in bottom panels. Scale bar = 20 μm. **G** Percentage of neurons with or without synaptopodin puncta in the AIS in the presence or absence of SMIFH2. **p < 0.01, Mann-Whitney test. **H** Percentage of synaptopodin puncta–positive neurons in the AIS of 21 DIV hippocampal neurons treated with 15 μM SMIFH2 in the presence or absence of 10 nM jasplakinolide for 3 h. ***p < 0.01, Mann-Whitney test. Data were acquired from 6 independent experiments and represented as the mean ± SEM. **I** Normalized ankyrinG intensity in 14 DIV hippocampal neurons treated for 3 h with 15 μM SMIFH2 alone or in combination with 10 nM Jasplakinolide **p < 0.01, ***p < 0.001, Kruskal-Wallis, Dunn’s multiple comparison test. Data were acquired from 3 independent experiments and represented as the mean ± SEM

Since formins remodel the cytoskeleton [38, 39], we analyzed actin and microtubule properties at the AIS after SMIFH2 treatment. First, we analyzed F-actin at the AIS in 14 DIV neurons treated for 3 h with DMSO or SMIFH2 (15 μM) (Fig. 3A). F-Actin was identified using phalloidin, and we observed a previously described actin patches staining at the AIS [16]. F-actin intensity was measured along the ankyrinG staining in confocal sections of control or SMIFH2-treated neurons (*n* = 165, Fig. 3B, C). Phalloidin staining showed a 20% reduction of F-actin all along the AIS (Fig. 3B) in SMIFH2-treated neurons (82 ± 2% vs 100 ± 2.26% in control neurons, Fig. 3C). Quantification of ankyrinG in the same neurons showed a 25% reduction in SMIFH2-treated neurons along the AIS (Fig. 3D). In contrast, ankyrinG intensity was not modified by inhibition of other important actin regulator, Arp2/3, using CK-666 (50 μM) for 3 h (Fig. 3E). Then, we analyzed the actin-related cisternal organelle at the AIS (Fig. 3F), using its characteristic synaptopodin staining [53]. Only 25 ± 5% of neurons treated with SMIFH2 were synaptopodin-positive at the AIS, compared to 90 ± 2% of DMSO-treated neurons (Fig. 3G). Actin stabilization by jasplakinolide (10 nM) pretreatment for 1 h increased the percentage of neurons with a detectable cisternal organelle (59 ± 5%) after SMIFH2 treatment (Fig. 3H). However, jasplakinolide did not prevent ankyrinG intensity reduction (Fig. 3E), suggesting that ankyrinG reduction due to formin inhibition involves an actin-independent mechanism.

**Fig. 4:**
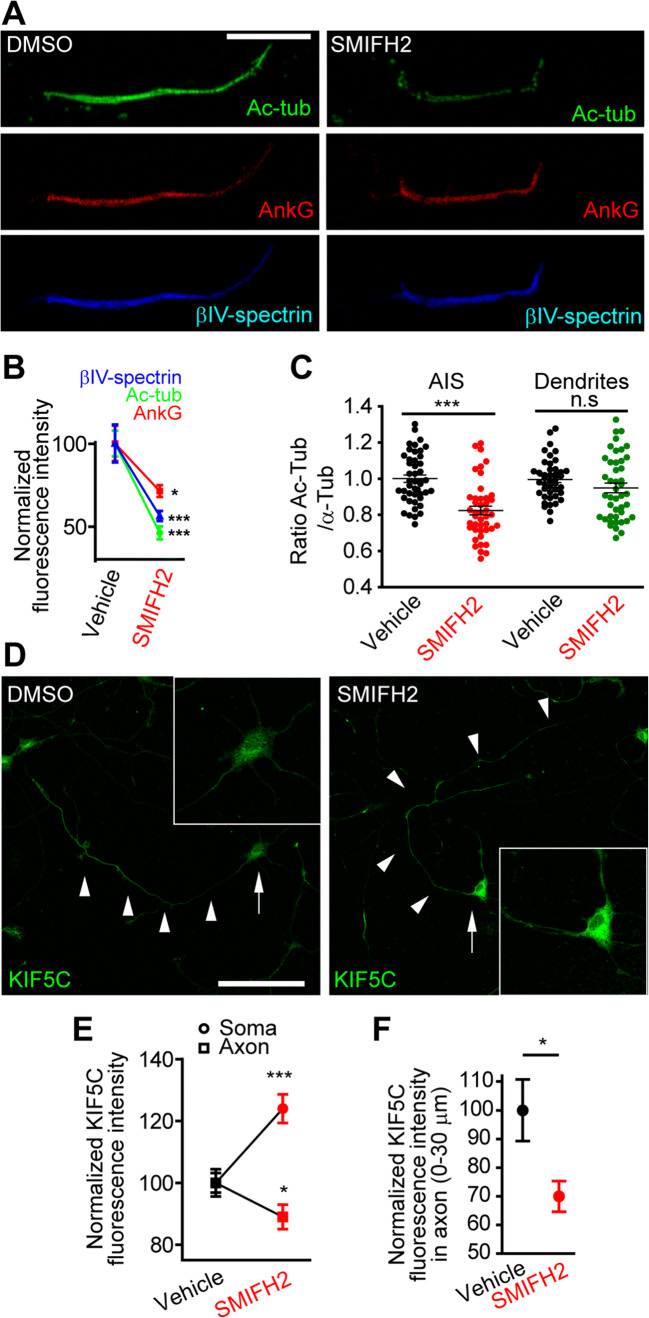
Formin inhibition modifies microtubules in the AIS and axonal transport. **A** Images of acetylated-tubulin (green), ankyrinG (red) and βIV-spectrin (blue) in the AIS of 13 DIV control and SMIFH2-treated neurons after detergent extraction for 5 min before fixation. Scale bar = 10 μm. **B** Normalized fluorescence intensity of markers indicated in **A**. *p<0.05, ***p < 0.001, Mann-Whitney test. **C** Acetylated-α-tubulin/α-tubulin ratio in the AIS or proximal dendrites in control or 15 μM SMIFH2-treated neurons. **D** KIF5C distribution in control or 15 μM SMIFH2-treated 5 DIV neurons for 3 h. Insets show magnifications of KIF5C expression in soma. Scale bar = 100 μm. **E** KIF5C normalized fluorescence intensity in soma (circles) and axon (squares) in DMSO (black) or SMIFH2 (red) treated neurons. *p < 0.05, ***p < 0.001, Mann-Whitney test. **F** KIF5C normalized fluorescence intensity in proximal axon of 5 DIV neurons treated with 15 μM SMIFH2 or DMSO. *p < 0.05, Mann-Whitney test. Data were acquired from three independent experiments and represented as the mean ± SEM

Thus, we analyzed whether formin inhibition was modifying microtubule characteristics leading to AIS protein density reduction. Actually, formins also contribute to increase microtubule acetylation and stability [39, 54]. Therefore, we quantified immunofluorescence intensity of acetylated-α-tubulin after detergent extraction in control and SMIFH2-treated neurons (Fig. 4A). SMIFH2-treated neurons showed a significant decrease in acetylated-α-tubulin staining (46 ± 8%) compared to 100 ± 4% in control neurons (Fig. 4B). AnkyrinG and βIV-spectrin signals were also significantly decreased (71 ± 4% and 56 ± 3% of the control level, respectively). Moreover, analysis of acetylated-α-tubulin vs total tubulin ratio without detergent extraction shows a significant reduction in the AIS (0.82 ± 0.02 vs 1.0 ± 0.02 in DMSO neurons) that was not found in proximal dendrites (Fig. 4C). Kinesin-1 selectively interacts with acetylated microtubules guiding this motor into the axon [19, 55]. Thus, we analyzed KIF5C distribution after SMIFH2 treatment. KIF5C showed a significant increase in 5 DIV neurons soma (124 ± 4.62%) accompanied by a reduction in axons (89 ± 4%) compared to DMSO-treated neurons (Fig. 4D, E). We detected a 30% KIF5C reduction in the first 30 microns of the axon compared to that in control cells (Fig. 4F). The above results suggest a participation of formin activity on AIS protein maintenance through a mechanism that involves microtubules.

### mDia1 Contributes to Maintain AIS Proteins

A previous study in neurons confirmed that mDia1 is the primary target of SMIFH2 activity on microtubules in hippocampal neurons [49]. Moreover, mDia1 is necessary to maintain neuroepithelial cell polarity [42] and modulates axon and dendrites structures [49, 56], being related to microtubule stability and acetylation, and actin dynamics modulation [54]. Thus, we stained 14 DIV hippocampal neurons with 3 different mDia1 antibodies (see “Methods” section), one of them validated in mDia1 knockout cells (Abcam). The staining pattern of all three mDia1 antibodies was similar and not specifically enriched at the AIS, though mDia1 staining appeared at the AIS (Fig. S4A-C). All three antibodies stained mainly the soma with a Golgi and perinuclear staining, as well as dendrites and axons (Fig. S4A–C). We observed a similar pattern in hippocampal neurons expressing CAmDia1-GFP (Fig. S4D). To analyze the potential role of mDia1 on AIS modulation, we used two different mDia1 interference RNAs (shmDia1-1, shmDia1-2) that express RFP protein as reporter (Fig. S5). Interference RNAs were validated in Neuro2a cells by immunofluorescence using the mDia1 knockout–validated antibody (Abcam). Both interference RNAs decreased mDia1 signal by 70%, 3 days after transfection, compared to cells transfected with scrambled shRNA (Fig. S5A–D).

**Fig. 5:**
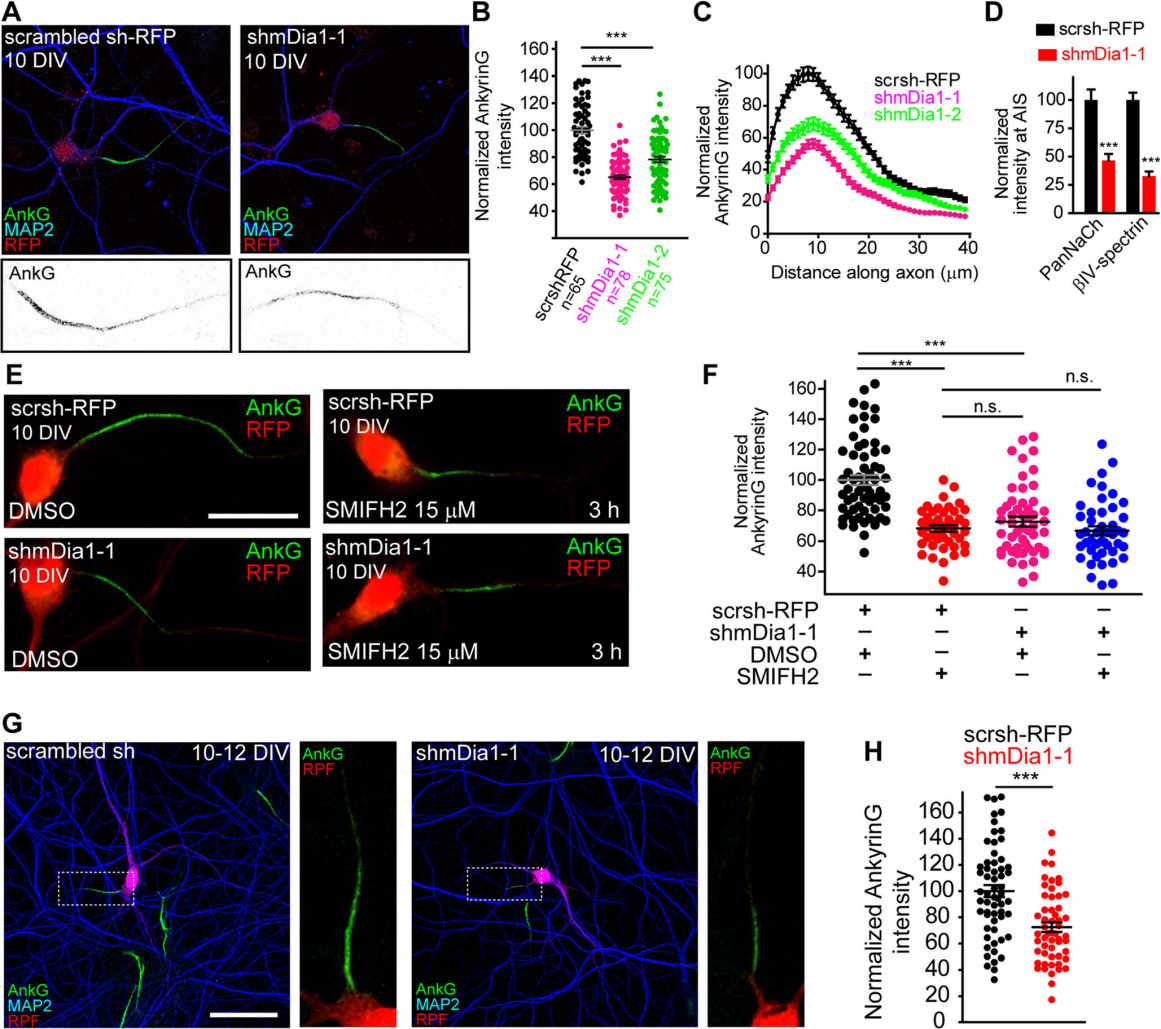
Suppression of mDia1 by interference shRNAs decreases AIS proteins accumulation. **A** Representative images of 10 DIV hippocampal neurons nucleofected with scrambled interference RNA (scrRFP) or mDia1 interference RNAs (shmDia1-1, shmDia1-2). RFP fluorescence identifies nucleofected neurons (red) and ankyrinG (green) the AIS. Bottom panels show AIS magnifications. Scale bar = 20 μm. **B** Normalized ankyrinG fluorescence intensity in the AIS of 10 DIV scrsh-RFP, shmDia1-1, or shmDia1-2 nucleofected hippocampal neurons. ***p < 0.001, one-way analysis of variance, Tukey’s multiple comparison test. **C** Normalized ankyrinG intensity profile along the AIS in 10 DIV-nucleofected neurons shown in **B**. **D** Normalized fluorescence intensity of voltage-gated sodium channels (PanNaCh) and βIV-spectrin in the AIS of 7 DIV-nucleofected hippocampal neurons. ***p < 0.001, Mann-Whitney test (*n* = 50 neurons). **E** AnkyrinG staining in 10 DIV scrsh-RFP- or shmDia1-1-nucleofected hippocampal neurons treated for 3 h with DMSO or 15 μM SMIFH2. Scale bar = 20 μm. **F** Normalized ankyrinG fluorescence intensity in the AIS of 10 DIV hippocampal neurons shown in **E**. n.s., not significant, ***p < 0.001, one-way analysis of variance, Tukey’s multiple comparison test. **G** 12 DIV hippocampal neurons lipofected with scrsh-RFP or shmDia1-1 plasmids at 10 DIV. Right panels show AIS magnifications and ankyrinG signal in transfected neurons. Scale bar = 50 μm. **H** Normalized ankyrinG fluorescence intensity 48 h after transfection of neurons shown in **G**, ***p < 0.0001, unpaired *t*-test (*n* = 60). Data in graphs were acquired from three independent experiments and represented as the mean ± SEM

To analyze mDia1 shRNA effects on neurons, first we checked that mDia1 shRNA was not affecting axon and dendrite development and did not find differences between scrambled shRNA- and shmDia1-1-nucleofected neurons regarding axon length at 3 DIV or dendrites length at 7DIV (Fig. S5G, H). Next, we nucleofected hippocampal neurons with shmDia1-1, shmDia1-2, or srcambled shRNA plasmids for 10 DIV and analyzed mDia1 staining in the soma of hippocampal neurons, where it is more densely localized (Fig. S5E–F). We found that mDia1 signal was reduced around 45% (shmDia1-1) and 30% (shmDia1-2) compared to scrambled shRNA-nucleofected neurons (Fig. S5F). Therefore, we analyzed ankyrinG expression at the AIS of 10 DIV neurons nucleofected with scrambled shRNA or mDia1 shRNAs (Fig. 5A, B). Both shmDia1-1 and shmDia1-2 shRNAs reduced significantly ankyrinG expression (Fig. 5B) to 68 ± 2% or 78 ± 2%, respectively, compared to scrambled shRNA (100 ± 2%). The higher reduction produced by shmDia1-1 compared to that by shmDia1-2 was in agreement with the previously observed higher mDia1 signal decrease (Fig. S5F). The AnkyrinG intensity profile along the AIS showed that this decline was homogenous all along the AIS (Fig. 5C). Moreover, 10 DIV shmDia1-1-nucleofected neurons showed a significant fluorescence intensity decrease of voltage-gated sodium channels and βIV-spectrin signals (47 ± 6% and 33 ± 4%, respectively, Fig. 5D). Besides that, ankyrinG intensity in shmDia1-1-nucleofected neurons was not further reduced after SMIFH2 (15 μM) treatment for 3 h (Fig. 5E, F) compared to that after DMSO treatment (67 ± 3% vs 73 ± 3%, respectively). However, SMIFH2 produced a significant ankyrinG reduction in scrambled shRNA-nucleofected neurons (68 ± 2% vs 100 ± 3% in DMSO-treated scrambled shRNA neurons), suggesting a SMIFH2 action through mDia1 inhibition. Next, we investigated whether mDia1 suppression in later developmental stages did also reproduce the ankyrinG reduction observed after SMIFH2 treatment. Neurons were transfected at 10 DIV with shmDia1-1 or scrambled shRNAs (Fig. 5G) and analyzed 2 days after. We found a significant ankyrinG reduction (72 ± 4%) in neurons transfected with shmDia1-1 plasmid compared to scrambled shRNA-transfected neurons (Fig. 5H). These data suggest that neuronal expression of mDia1 contributes to the maintenance of AIS proteins from early developmental to mature stages of AIS.

### AIS Maintenance by Formins Involves Microtubule-Related Mechanisms

**Fig. 6:**
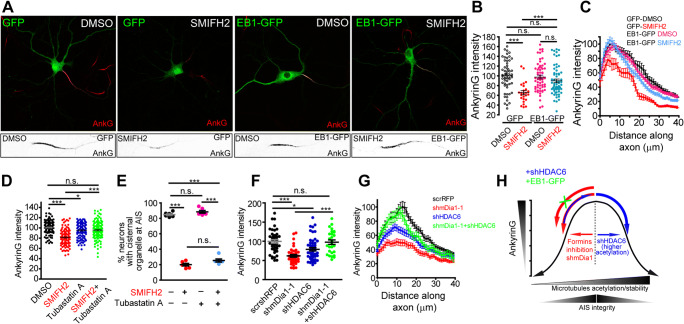
Increased microtubule acetylation and stabilization impairs formin inhibition–mediated ankyrinG loss. **A** Representative images of 11 DIV hippocampal neurons transfected with GFP or EB1-GFP for 48 h and then exposed to DMSO or 15 μM SMIFH2 for 3 h. AIS and ankyrinG magnifications are shown in bottom panels. **B** Normalized ankyrinG fluorescence intensity of neurons shown in A. n.s., not significant, **p < 0.01, ***p < 0.001, Kruskal-Wallis, Dunn’s multiple comparison test. **C** AnkyrinG profile along the proximal axon of neurons shown in **B**. **D** Normalized ankyrinG intensity in 14 DIV neurons treated with DMSO or 15 μM SMIFH2 alone or in combination with the tubulin deacetylase HDAC6 inhibitor Tubastatin A for 3 h. **E** Percentage of neurons with detectable cisternal organelle after Tubastatin A, SMIFH2, or their combination. Data represent the mean ± SEM obtained from 6 independent experiments. **F** Normalized ankyrinG intensity in 7 DIV hippocampal neurons nucleofected with shmDia1-1, shHDAC6, or their combination. Scrsh-RFP nucleofected neurons were used as control to normalize data. n.s., not significant, *p < 0.05, ***p < 0.001, Kruskal-Wallis, Dunn’s multiple comparison test. **G** AnkyrinG profile along the proximal axon of neurons shown in **F**. Data were acquired from three independent experiments and represented as the mean ± SEM. **H** Schematic representation summarizing the role of formins in the regulation of ankyrinG levels. Decreased formin activity leads to lower acetylation and stability that is counteracted by increasing microtubule acetylation through shHDAC6 or stability by EB1. High tubulin acetylation by shHDAC6 alone also decreases ankyrinG levels

Since our results demonstrate that formin inhibition decreases tubulin acetylation on AIS microtubules, we hypothesized that ankyrinG decrease could be prevented increasing microtubule stability/acetylation. The microtubule end-binding protein, EB1, binds to AIS microtubules [3], interacts with mDia1 and other mDia proteins, and contributes to stabilize microtubules [57, 58]. Moreover, EB1 allows ankyrinG association to microtubules plus ends [13]. Thus, we expressed EB1-GFP protein in 10 DIV hippocampal neurons before SMIFH2 treatment at 12 DIV (Fig. 6A, B, C). AnkyrinG fluorescence intensity decrease observed in GFP control neurons after SMIFH2 treatment (65% ± 4%, Fig. 6B, C) was significantly prevented in EB1-GFP transfected neurons treated with SMIFH2 (90% ± 4% ; *p* < 0.0001, *t*-test, Mann-Whitney test) and happened all along the AIS (Fig. 6C). We did not detect any significant change between GFP:DMSO (100 ± 4%), EB1-GFP:DMSO (99 ± 4%), and EB1-GFP:SMIFH2 neurons (*p* = 0.07, Kruskal-Wallis test). Then, we tested whether increasing microtubule acetylation maintains ankyrinG levels after formin inhibition or reduction of mDia1 expression. For that purpose, we inhibited the tubulin deacetylase HDAC6 using Tubastatin A (10 μM). The ankyrinG reduction (84 ± 2%) produced by SMIFH2 after 3 h in 14 DIV neurons was prevented by the HDAC6 inhibitor co-treatment (106 ± 3%) compared to 100 ± 2% in control neurons (Fig. 6D). HDAC6 inhibition alone also reduced ankyrinG intensity, as previously described [21]. However, Tubastatin A did not recover the actin-related cisternal organelle loss due to SMIFH2 inhibition (Fig. 6E), in opposition to cisternal organelle recovery in jasplanikinolide-SMIFH2 co-treated neurons (Fig. 3H), suggesting a formin-microtubule role on ankyrinG maintenance. We next analyzed whether HDAC6 interference RNA (shHDAC6) could impair ankyrinG reduction in neurons expressing mDia1 interference RNA (Fig. 6F, G). As happened after HDAC6 inhibition, shHDAC6 reduced ankyrinG intensity by 20% due to higher general tubulin acetylation, as previously described [21, 22]. This reduction was higher all along the AIS in neuron-expressing shmDia1-1 (62 ± 4%, Fig. 6F, G). However, neurons co-expressing shmDia1-1 and shHDAC6 interference RNAs showed no ankyrinG change (98 ± 6%) compared to 100 ± 3% in control scrambled shRNA neurons (Fig. 6F), suggesting a potential compensation of tubulin acetylation that tends to maintain control levels as represented in Figure 6H.

### Formin Inhibition or mDia1 Suppression Shortens the AIS

**Fig. 7:**
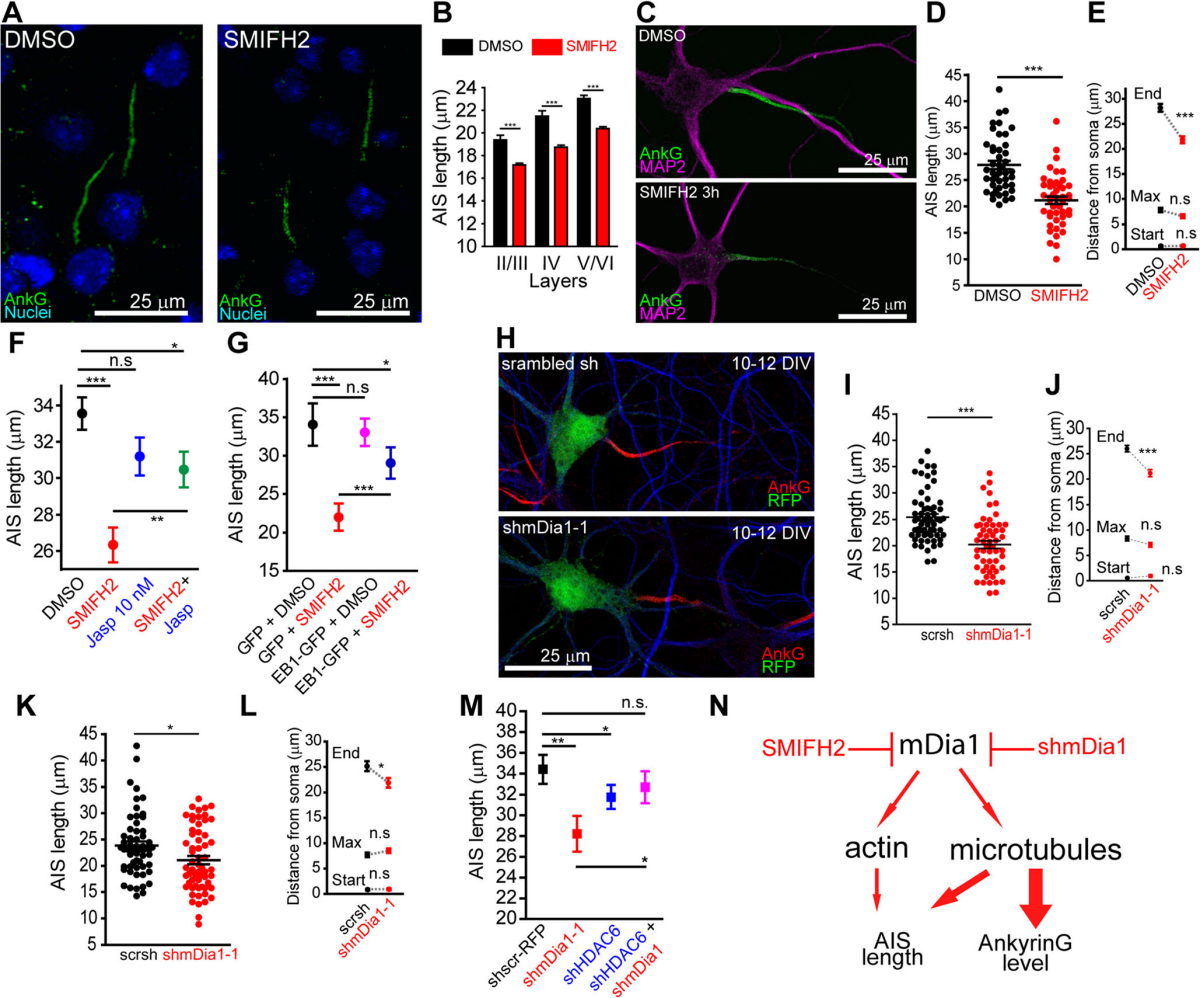
Decreased formin activity leads to AIS shortening prevented by actin and microtubule stabilization. **A** Representative images of AISs (green) in layer IV of P30 mice cortical sections treated with DMSO or 30 μM SMIFH2. Nuclei are stained with bis-benzimide (blue). Scale bar = 25 μm. **B** Quantification of AIS length, in 150 neurons from each layer in 3 independent experiments, in the presence (red) or absence (black) of 30 μM SMIFH2 in layers II/III, IV, or V/VI. ***p < 0.0001, Mann-Whitney test. **C** Representative images of AISs in 10 DIV neurons treated with DMSO or SMIFH2 15 μM for 3 h. **D** Quantification of AIS length in DMSO- or SMIFH2-treated neurons shown in **C** (60 neurons from 3 experiments). **E** Mean ± SEM start, maximum, and end position in DMSO- or SMIFH2-treated neurons. **F** AIS length in neurons treated with SMIFH2 (15 μM) and/or jasplakinolide (10 nM), *n* = 80 neurons from 3 experiments. (**G** AIS length in GFP- or EB1-GFP-lipofected neurons treated with SMIFH2 (15 μM) or DMSO (60 neurons from 3 experiments). **H** Representative images of AISs in 12 DIV shmDia1-1- or scrsh-RFP-transfected neurons for 48 h. **I–L** Quantification of AIS length in 12 DIV shmDia1-1–transfected neurons (**I**) and 10 DIV-nucleofected neurons (**K**). Mean ± SEM start, maximum, and AIS end position of these neurons are shown in **I** and **K**, respectively. **M** Quantification of AIS length in 12 DIV neurons transfected with shmDia1-1 and/or shHDAC6 plasmids for 48 h. Data are from 3 independent experiments and 40 neurons by condition. Data are represented as the mean ± SEM. n.s., not significant, *p < 0.05, **p < 0.01, ***p < 0.0001, Mann-Whitney test. **N** Schematic representation that summarizes the role of formins in the regulation of ankyrinG levels and AIS length. Reduced formin activity or mDia1 actin modifications only affect AIS length, while microtubule modifications reduce AIS length and ankyrinG levels

Next, we analyzed how formin inhibition or mDia1 downregulation may produce changes on AIS length or position. We first analyzed AIS length in brain slices treated with SMIFH2 (30 μM) for 3 h (Fig. 7A, B). We found that AIS length was significantly different between cortical layers in P30 female mice (19.39 ± 0.41 μm in layers II/III, 21.49 ± 0.47 μm in layer IV and 23.04 ± 0.28 μm in layers V/VI) and analyzed this parameter by layer groups after SMIFH2 treatment (Fig. 7B). Formin inhibition produced a similar and significant AIS length reduction, around 12%, in each layer group (Fig. 7B). SMIFH2 (15 μM) treatment in 10 DIV hippocampal neurons for 3 h reduced 20% of the AIS length (21.15 ± 0.72 μm compared to 27.86 ± 0.79 μm in DMSO-treated neurons, Fig. 7C, D, E). Despite that actin stabilization did not recover ankyrinG levels, pre-incubation with jasplakinolide 1 h before SMIFH2 treatment partially prevented AIS shortening (Fig. 7F). We observed the same partial AIS shortening prevention in neurons expressing EB1-GFP after SMIFH2 treatment (Fig. 7G). Transfection of shmDia1-1 plasmid (Fig. 7H, I, J) in 10 DIV hippocampal neurons did also reduce a 20% the AIS length after 2 days (20.21 ± 0.72 μm vs 25.43 ± 0.63 μm in scrambled shRNA neurons). A similar AIS length reduction (15%) happened in shmDia1-1-nucleofected neurons (Fig. 7K, L) after 10 DIV (21.09 ± 0.78 μm vs 23.85 ± 0.77 μm in scrambled shRNA-nucleofected neurons). AIS starting position or the maximum intensity position did not change in SMIFH2-treated neurons or neuron-transfected or nucleofected with shmDia1-1 plasmid (Fig. 7E, J, L) proving a distal shortening of the AIS. Finally, we analyzed whether shHDAC6 expression, shown to recover ankyrinG density (Fig. 6F), could prevent mDia1 shRNA–mediated AIS shortening (Fig. 7M). AISs were again shorter in shmDia1-1-transfected neurons (28.25 ± 1.71 μm vs 34.41 ± 1.40 μm in scrambled shRNA neurons), while those expressing shHDAC6 and shmDia1-1 interference RNAs had AISs not significantly shorter (32.69 ± 1.53 μm). Neurons only expressing shHDAC6 had slightly but significantly shorter AISs (31.19 ± 1.05 μm). From our results, we propose a dual role of formins in AIS actin and microtubules, where microtubule stability/acetylation contributes to maintain mainly ankyrinG density and AIS length, while actin modulation is only involved in AIS length regulation (Fig. 7N).

## Discussion

Axon initial segment plays an important role in the control of intrinsic neuronal excitability, maintains neuronal polarity, and is capable of a high and dynamic structural plasticity [9, 59]. These important roles require not fully understood mechanisms that modulate AIS cytoskeleton. In our study, we hypothesized that formins, as important cytoskeleton regulators [34, 39] that participate in different cellular mechanisms and cell polarity [60], may regulate AIS cytoskeleton contributing to its maintenance and plasticity.

We report that formin activity is necessary to maintain structural ankyrinG and βIV-spectrin protein or sodium channel density at the AIS. A short time inhibition of formins, using 15 μM SMIFH2 concentration lower than the IC_50_ (28 μM) previously described in 3T3 fibroblasts [51], is enough to decrease ankyrinG density, both in vitro and ex vivo. We also detected a myosin II light chain phosphorylation (pMLC) decrease, previously identified in relation to AIS plasticity [14], and a recent study proposed a SMIFH2 effect on myosins [61] in filopodia adhesion. However, even high SMIFH2 concentrations up to 100 μM do not affect non-muscle myosin II phosphorylation [62], suggesting a decrease due to AIS structural protein modifications. In fact, although SMIFH2 (30 μM) effects are reversible upon washout on fibroblasts in a short time [51], we found that SMIFH2-induced modifications after 3 h are not reversible in neurons and led to a later loss of axonal polarity, as previously described after ankyrinG knockdown [1]. Polarity loss happens also in mDia-deficient mice that show a disrupted integrity of their neuroepithelium [42], while in epithelial cells, mDia1 knockdown do not affect the formation of tight junctions, but their protein content decreases and epithelial barrier is compromised [40] as observed in our study in neurons. The effects produced by SMIFH2 could be associated to different formins expressed in neurons, as it is a general formin inhibitor [51]. However, we show that two different mDia1 shRNAs also mimic SMIFH2-mediated ankyrinG, βIV-spectrin, and sodium channel density decrease, maintaining a correlation between mDia1 staining reduction in soma and ankyrinG decrease. These results are in line with previous results in neurons demonstrating that mDia1 is the primary target of SMIFH2 [49] and our results showing any further change in ankyrinG density after SMIFH2 treatment in neurons expressing mDia1 interference RNA.

Previous studies identified mDia1 in dendrites [49], Golgi, or axonal growth cones [44]. mDia1 antibodies used in this study, including one validated in knockout cells, detected mDia1 staining at the AIS. However, most mDia1 antibodies signal and CAmDia1-GFP signal were detected in soma, dendrites, and axons opening the possibility that SMIFH2 effects on AIS composition and structure may come from changes in these structures. Some studies suggest that higher AIS protein density correlates with a more complex dendritic arbor [63, 64]. Previous studies have shown that mDia1 interference RNAs reduce dendritic ramification in 18 DIV hippocampal neurons [65], while a higher ramification index is observed in 21 DIV cortical neurons after 7 days without affecting spine density [49]. We did not find any significant change in dendrite length after 7 DIV in neurons nucleofected with mDia1 shRNAs. Thus, ankyrinG decrease found in our study does not seem to be related to changes in dendrites, although we cannot completely discard an effect due to presynaptic or postsynaptic activity changes that may affect AIS structure and composition. Indeed, mDia1 suppression do not alter spine density or modify mEPSC frequency or amplitude [49] in physiological conditions, but SMIFH2 (30 μM)-mediated formin inhibition dramatically disrupts synaptic recycling in hippocampal neurons [43], what can modify neuronal inputs and generate changes on AIS structure.

Our data show changes in AIS actin and microtubule cytoskeleton after formin inhibition. Indeed, formins are important regulators of actin nucleation and elongation [66], and regulate microtubule dynamics and stability [57, 67]. We found that formin inhibition was able to disrupt actin filaments and cisternal organelle. Actin cytoskeleton stabilization by jasplakinolide did partially impaired cisternal organelle loss and AIS shortening, but did not impair ankyrinG density decrease. Thus, formin inhibition–mediated actin modifications seem to play only a minor role on AIS length, due to cisternal organelle alterations, previously shown to play a role in AIS length plasticity [27]. Moreover, actin rings do not change during AIS developmental reorganization and shortening [68]. In this sense, mDia1 modulates actin-independent mechanisms, such as the spine density recovery, after Aβ peptide treatment, by an actin-binding–deficient K994A mDia1 mutant [67]. We found that formin inhibition decreased tubulin acetylation in AIS microtubules, and not in proximal dendrites, which correlated with ankyrinG decrease. The AIS contains stable microtubules characterized by high detyrosinated and acetylated-tubulin levels compared to dendrites [19, 21], which influences the binding and motility of kinesin-1. Indeed, mDia1 contributes to the control of microtubule dynamics and increases tubulin acetylation [54], while decreased mDia1 expression leads to lower detyrosinated tubulin levels [49]. This explains the reduced kinesin-1 localization to the axon and ankyrinG density after formin inhibition. However, ankyrinG density and AIS length remain to control levels when the tubulin deacetylase HDAC6 is inhibited or knockdown in SMIFH2-treated neurons or neurons expressing mDia1 shRNA. HDAC6 knockdown also decreases AIS length and ankyrinG density, as previously studied [21], to a lesser extent than mDia1 knockdown. A suitable explanation is that both decreased or increased tubulin acetylation modify AIS integrity and lower ankyrinG density. Formin inhibition reduces tubulin acetylation, but HDAC6 suppression increases tubulin acetylation and compensate ankyrinG loss (Fig. 6H). Actually, HDAC6 inhibition compensates transport deficits in Huntington’s disease promoting kinesin-1 recruitment by increasing microtubule acetylation [69]. Previous studies showed that HDAC6 and mDia formins collaborate to modulate cytoskeleton [70]. In fact, HDAC6 cooperates with Dia1 in the formation of links between actin filaments and microtubules, affecting microtubule dynamics [71]. Besides, HDAC6 can physically associate to EB1 [72] that has been proposed to function downstream of mDia1 in microtubule stabilization [58] and can explain ankyrinG density and AIS length maintenance in neurons over-expressing EB1 when formins are inhibited. Moreover, EB1 not only participates in microtubule dynamics regulation [58, 73], but also is a component of the AIS [3], binding ankyrinG to microtubules [74].

AnkyrinG serves as the anchoring scaffold for voltage-gated sodium channels which density also decreased. In effect, lack of formin activity led to a reduced intrinsic neuronal excitability compatible with a sodium channel density decrease. Despite that the spike number is decreased, spike amplitude was not modified, which can be explained by the higher sensitivity of spike frequency compared to the mild reduction in sodium channel density (~20%, Fig. 1E) [75] observed after formin inhibition. In addition, formin inhibition depolarized the spike threshold, both in hippocampal and in cortical neurons. Such spike threshold increase may result from the reduction in sodium channel density or AIS length decrease due to the disorganization of the AIS by a formin inhibitor. In fact, a lower spike threshold has been associated to sodium channel density increase during AIS development in nucleus magnocellularis (NM) neurons [68]. Otherwise, spike threshold depolarization happens after AIS shortening due to blast wave exposure without any change in protein density [76]. A recent work suggests a differential regulation of sodium channel accumulation and AIS cytoskeleton reorganization, which work synergistically to optimize neuronal output [68].

In conclusion, our study describes a role of formins, and specifically mDia1, in the maintenance of AIS protein density and AIS length because of its direct or indirect action on AIS microtubules, but to a lesser extent on AIS actin-related structures (Fig. 7N). Formin loss of function or mutation is a characteristic in several mental disorders, as well as in neurodegenerative diseases [44]. Further studies are necessary to understand which physiological or pathological stimulus can modulate neuronal cytoskeleton through formins or mDia1 at the AIS or other neuronal compartments in order to regulate AIS structural plasticity.

## Supplementary Information

Supplementary Figure 1. 14 DIV hippocampal neurons treated for 3 h with DMSO (left panels) or 15 μM SMIFH2 (right panels). Neurons were stained with βIV-spectrin, PanNaCh (voltage gated sodium channels) or pMLC antibodies (green). Somatodendritic compartment was identified by MAP2 staining (red). Panels under each image show AIS magnifications. (PNG 25497 kb)

High Resolution Image (TIF 2179 kb)

Supplementary Figure 2. (**A**) AnkyrinG decrease due to formins inhibition is independent of potential SMIFH2 effects on astrocytes. Graph represents the normalized ankyrinG fluorescence intensity in 14 DIV hippocampal neurons cultured in the presence of astrocytes and transferred to plates containing neuronal medium conditioned by astrocytes for SMIFH2 15 μM treatment for 3 hours. (**B**) AnkyrinG intensity in 14 DIV neurons treated with DMSO or 15 μM SMIFH2 for 3 h, and maintained for another 6 h in fresh plates containing astrocytes. n.s., not significant, *p < 0.05, ***p < 0.001, Kruskal-Wallis, Dunn’s multiple comparison test. All data were acquired from three independent experiments, and at least 150 neurons, and represented as the mean ± SEM. (**C**) 14 DIV neurons treated with SMIFH2 (15 μM) and kept with fresh astrocytes for 24 hours to analyze MAP2 intensity at the AIS. MAP2 and AnkG staining are shown in grey in bottom panels. (**D**) MAP2 intensity profile was calculated in the first 50 μm of the axon in DMSO (black dots) or SMIFH2 treated neurons (red dots). (**E**) Correlation between ankyrinG and MAP2 in every neuron. (PNG 25497 kb)

High Resolution Image (TIF 1093 kb)

Supplementary Figure 3. **AnkyrinG decrease is not mediated by a calcium-calpain mechanisms after SMIFH2 treatment.** The graph represents the normalized ankyrinG fluorescence intensity of 14 DIV hippocampal neurons treated with the calpain inhibitor MDL-28170 (50 nM) 1 hour prior to SMIFH2 (15 μM) for 3 hours. n.s., not significant, ***p<0.001, Mann-Whitney test. (PNG 25497 kb)

High Resolution Image (TIF 162 kb)

Supplementary Figure 4. **mDia1 expression in hippocampal neurons** (**A-C**) 14 DIV hippocampal neurons stained with 3 different mDia1 antibodies (PA5-27607, 610849, and the knockout validated antibody ab129167). AIS was identified by ankyrinG signal and dendrites by MAP2 staining. AIS region and mDia1 staining or ankyrinG stainings are magnified in bottom panels. Scale bar = 20 μm. (**D**) Two examples of 12 DIV neurons expressing GFP-CA-mDia1 (green) after transfection at 10 DIV. Neurons were stained with ankyrinG (red) and MAP2 antibodies (blue in upper neuron). (PNG 25497 kb)

High Resolution Image (TIF 6124 kb)

Supplementary Figure 5. (**A-C**) Neuro2a cells transfected with srambled (A), shmDia1-1 (B) or shmDia1-2 (C) interference shRNAs for 3 days. Transfected cells are identified by RFP signal (red) and mDia1 knockout validated antibody (ab129167, Abcam) signal is show in green. (**D**) Quantification of mDia1 signal in transfected (red bars) or non-transfected (black bars) Neuro2a cells (n=50 cells/experimental condition). Data are represented as the mean ± SEM. n.s., not significant, ** p < 0.01***, p < 0.0001, Mann-Whitney test. (**E**) 10 DIV hippocampal neurons nucleofected with scrambled interference RNA (scrsh-RFP) or mDia1 interference RNAs (shmDia1-1, shmDia1-2). Nucleofected neurons were identified based on RFP fluorescence (magenta). Neurons were stained with mDia1 (green) and MAP2 (blue) antibodies. Scale bar = 20 μm. (**F**) mDia1 fluorescence intensity was quantified in the soma of neurons nucleofected with scrsh-RFP, shmDia1-1 or shmDia1-2 plasmids. Inserts in D show magnifications of mDia1 staining in soma. **p < 0.01, ***p < 0.001, One-way analysis of variance, Tukey’s multiple comparison test. Data in graphs were acquired from three independent experiments and represented as the mean ± SEM. (**G, H**) Axonal and dendritic growth is not affected by mDia1 interference RNA. Hippocampal neurons were nucleofected before plating with shmDia1-1 interference RNA and kept for 3 DIV (G) to analyze axonal length using the tau1 axonal marker, or 7 DIV to analyze total dendritic length using the MAP2 somatodendritic marker (H). (PNG 25497 kb)

High Resolution Image (TIF 1333 kb)

## Data Availability

Data generated and analyzed during this study are included in the published article and its supplementary information files.
