# Supplementary figures and images for: Formin Activity and mDia1 Contribute to Maintain Axon Initial Segment Composition and Structure
Source: Mol Neurobiol. 2021 Aug 30;58(12):6153–69. doi: 10.1007/s12035-021-02531-6 (PMC8639558; doi:10.1007/s12035-021-02531-6)

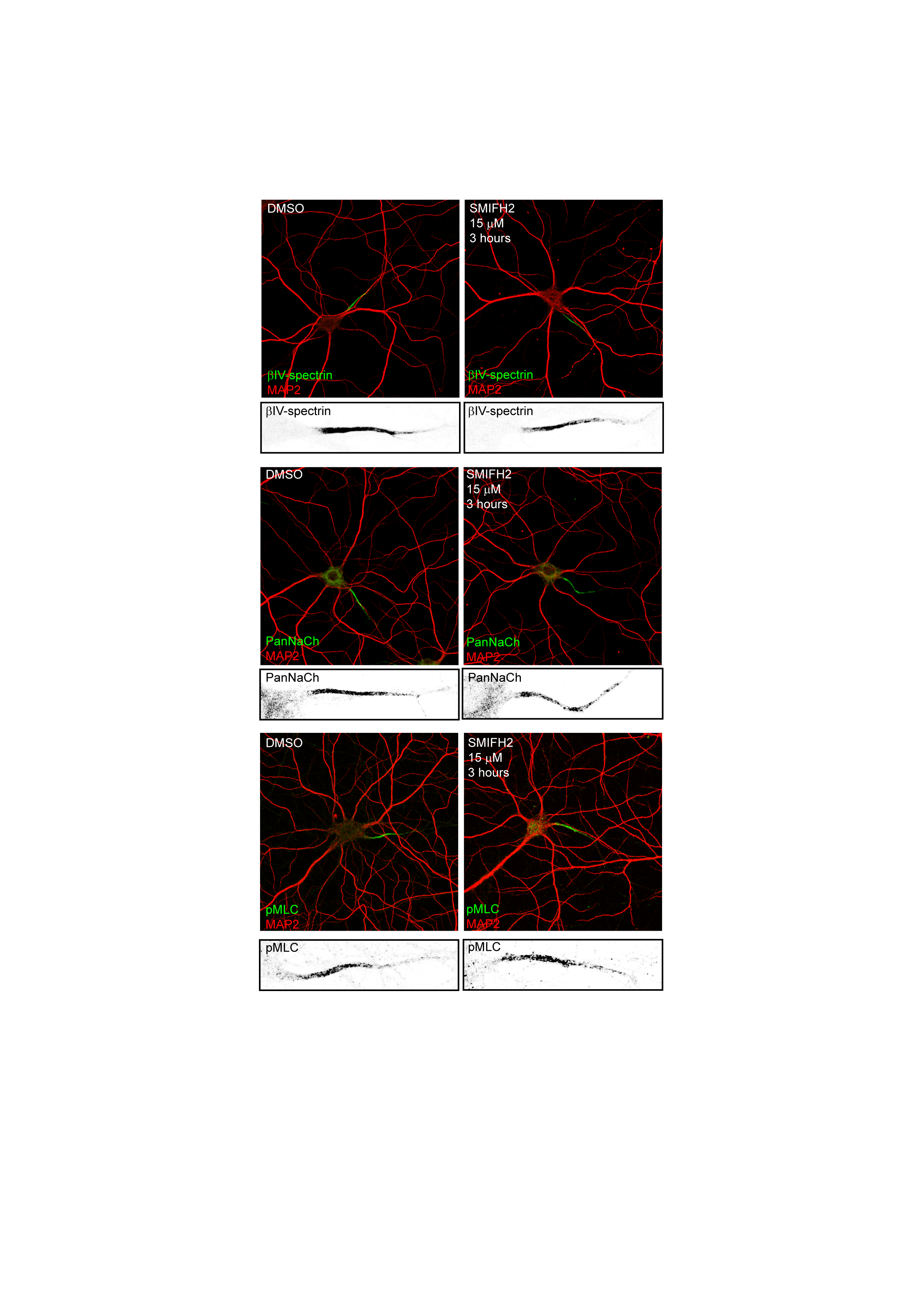

Supplement: Supplementary file 1 — 14 DIV hippocampal neurons treated for 3 h with DMSO (left panels) or 15 μM SMIFH2 (right panels). Neurons were stained with βIV-spectrin, PanNaCh (voltage gated sodium channels) or pMLC antibodies (green). Somatodendritic compartment was identified by MAP2 staining (red). Panels under each image show AIS magnifications. (PNG 25497 kb) [file 12035_2021_2531_Fig8_ESM.png]

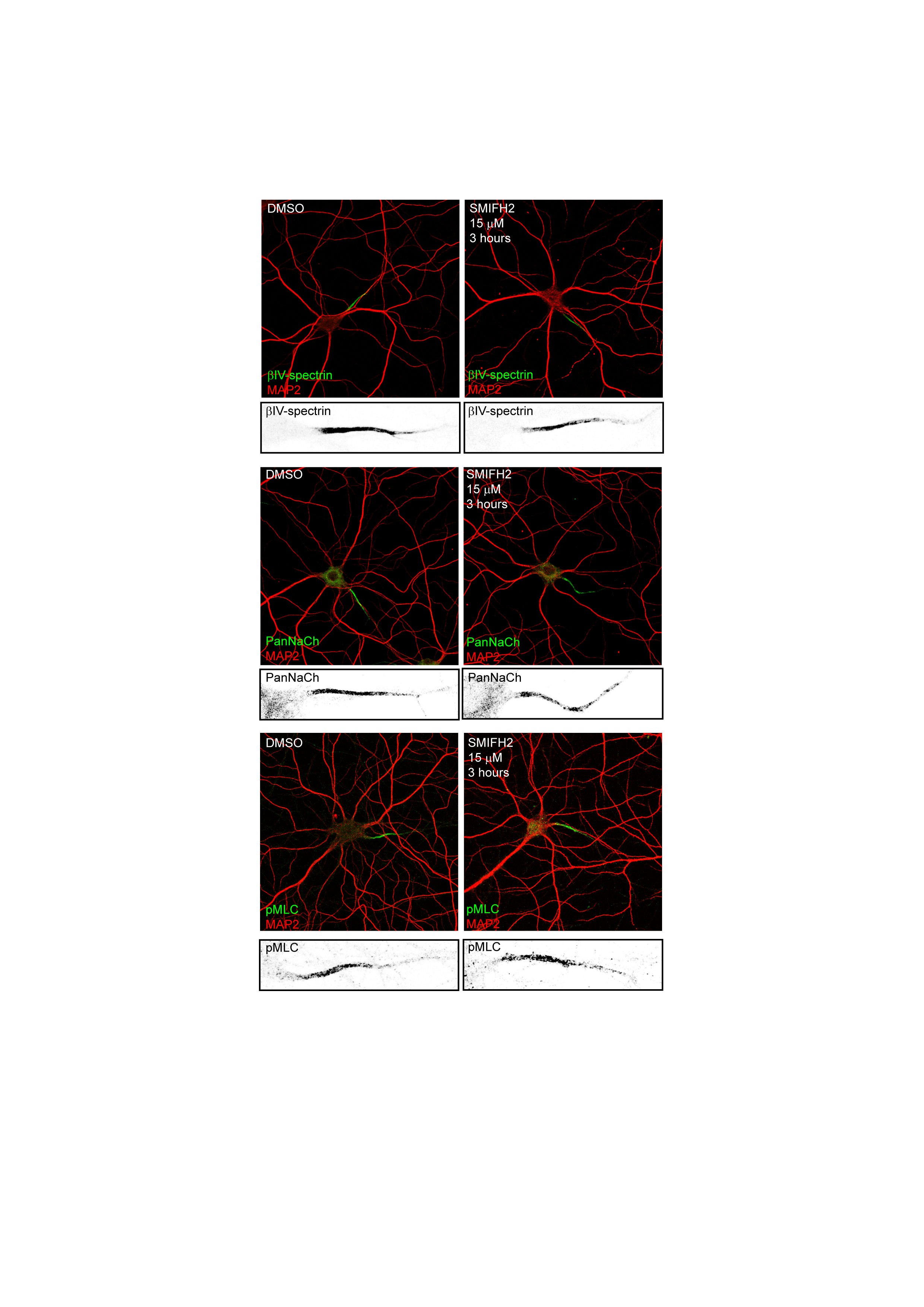

Supplement: Supplementary file 2 — High Resolution Image (TIF 2179 kb) [file 12035_2021_2531_MOESM1_ESM.tif]

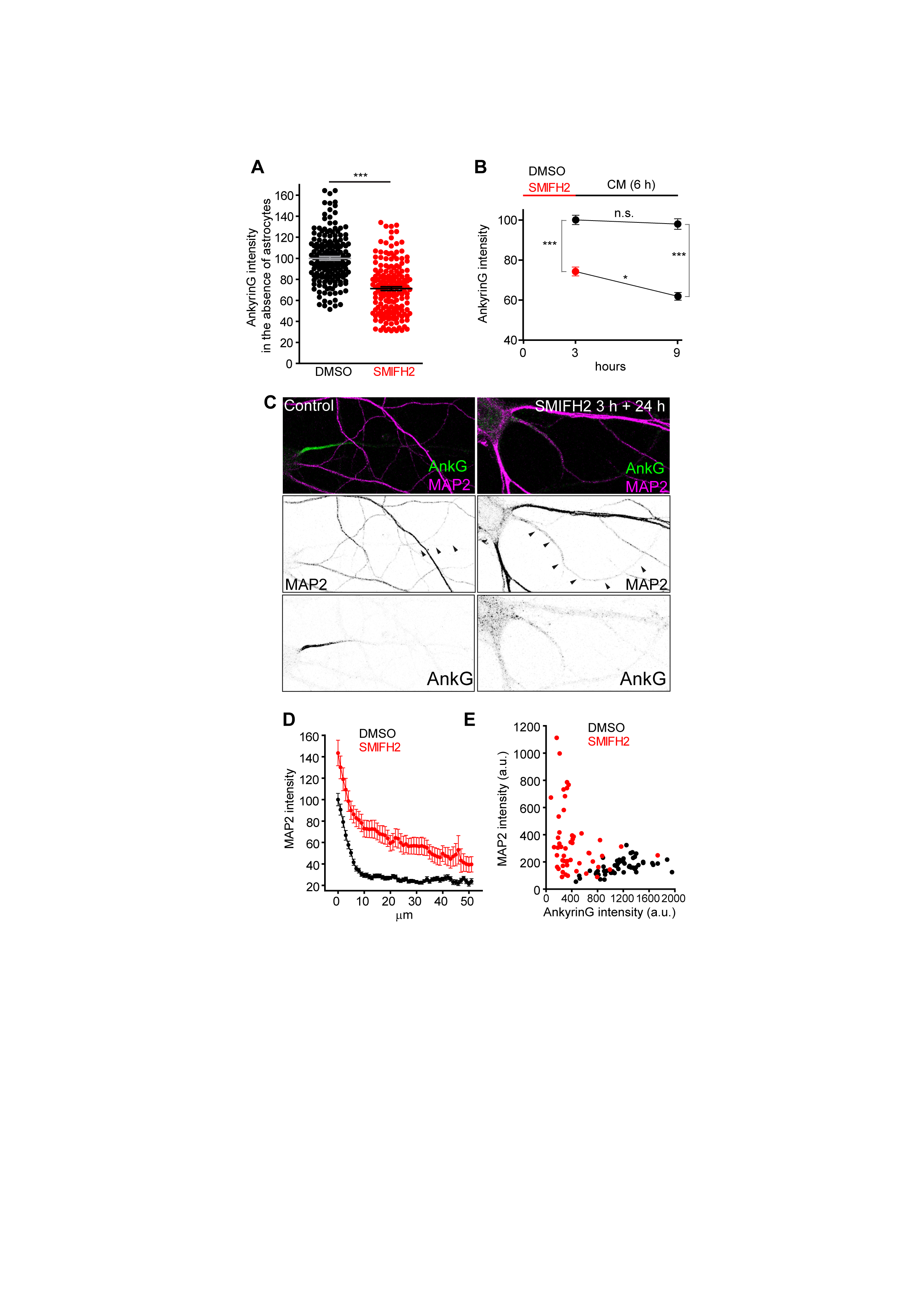

Supplement: Supplementary file 3 — (A) AnkyrinG decrease due to formins inhibition is independent of potential SMIFH2 effects on astrocytes. Graph represents the normalized ankyrinG fluorescence intensity in 14 DIV hippocampal neurons cultured in the presence of astrocytes and transferred to plates containing neuronal medium conditioned by astrocytes for SMIFH2 15 μM treatment for 3 hours. (B) AnkyrinG intensity in 14 DIV neurons treated with DMSO or 15 μM SMIFH2 for 3 h, and maintained for another 6 h in fresh plates containing astrocytes. n.s., not significant, *p < 0.05, ***p < 0.001, Kruskal-Wallis, Dunn’s multiple comparison test. All data were acquired from three independent experiments, and at least 150 neurons, and represented as the mean ± SEM. (C) 14 DIV neurons treated with SMIFH2 (15 μM) and kept with fresh astrocytes for 24 hours to analyze MAP2 intensity at the AIS. MAP2 and AnkG staining are shown in grey in bottom panels. (D) MAP2 intensity profile was calculated in the first 50 μm of the axon in DMSO (black dots) or SMIFH2 treated neurons (red dots). (E) Correlation between ankyrinG and MAP2 in every neuron. (PNG 25497 kb) [file 12035_2021_2531_Fig9_ESM.png]

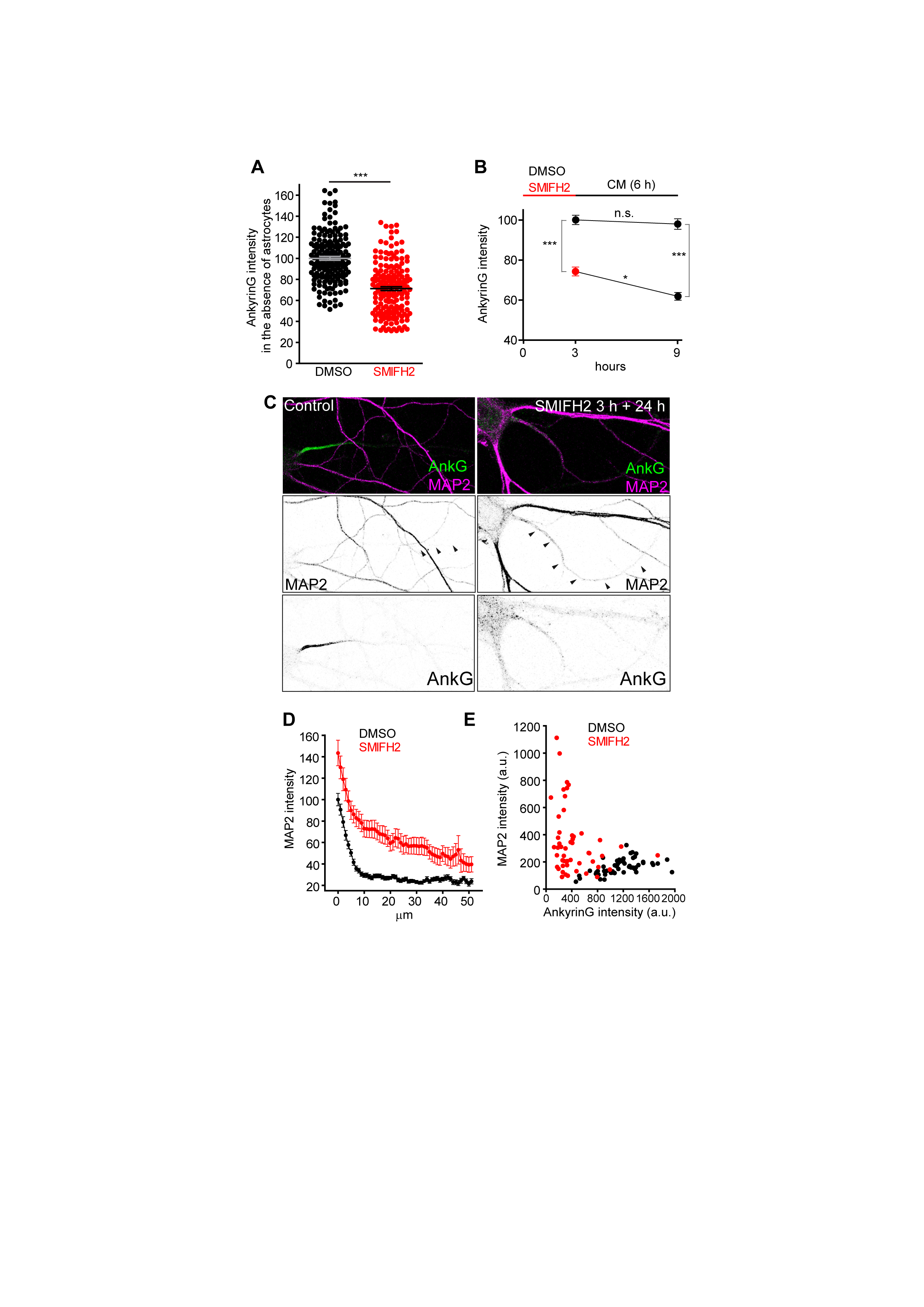

Supplement: Supplementary file 4 — High Resolution Image (TIF 1093 kb) [file 12035_2021_2531_MOESM2_ESM.tif]

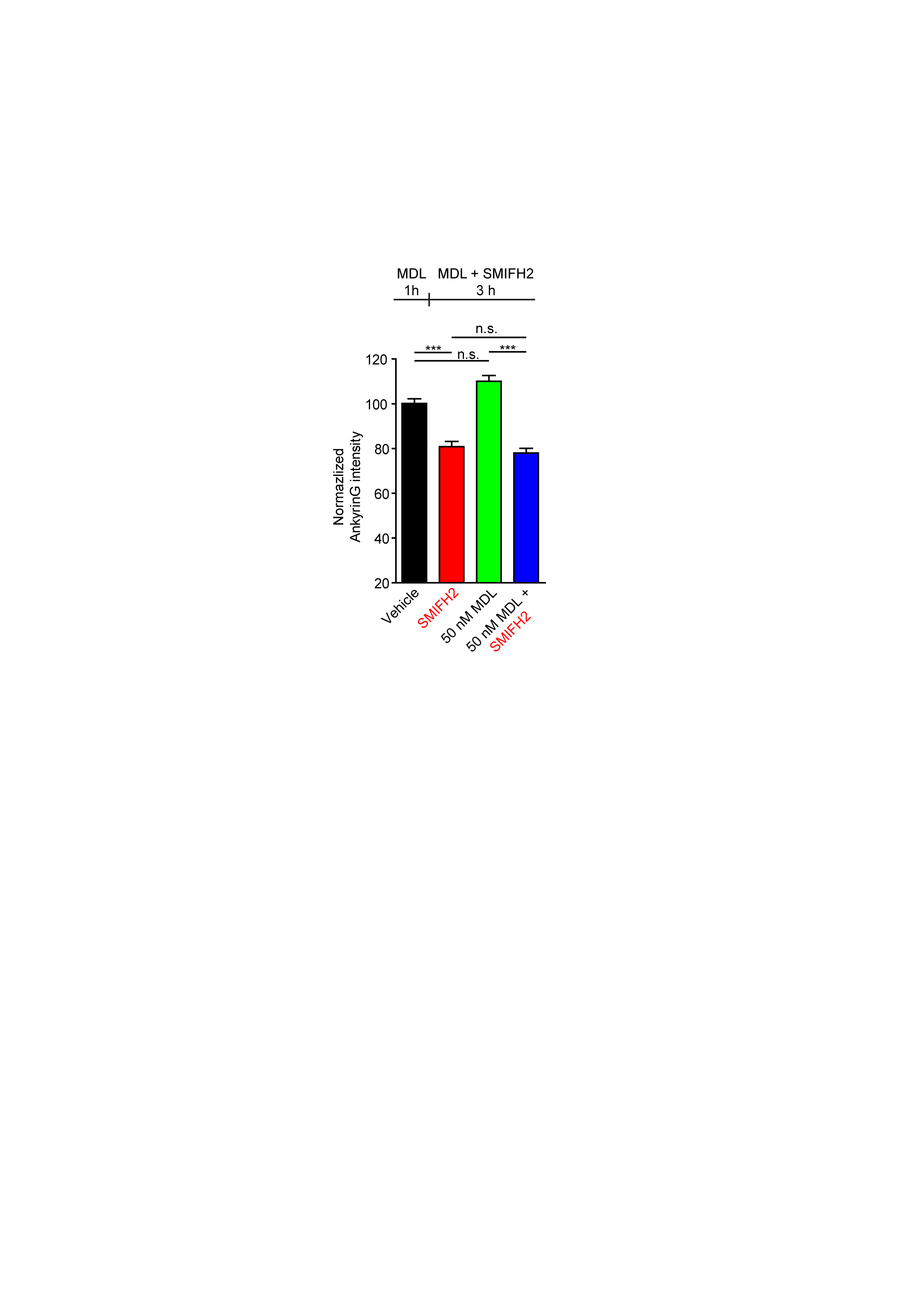

Supplement: Supplementary file 5 — AnkyrinG decrease is not mediated by a calcium-calpain mechanisms after SMIFH2 treatment. The graph represents the normalized ankyrinG fluorescence intensity of 14 DIV hippocampal neurons treated with the calpain inhibitor MDL-28170 (50 nM) 1 hour prior to SMIFH2 (15 μM) for 3 hours. n.s., not significant, ***p<0.001, Mann-Whitney test. (PNG 25497 kb) [file 12035_2021_2531_Fig10_ESM.png]

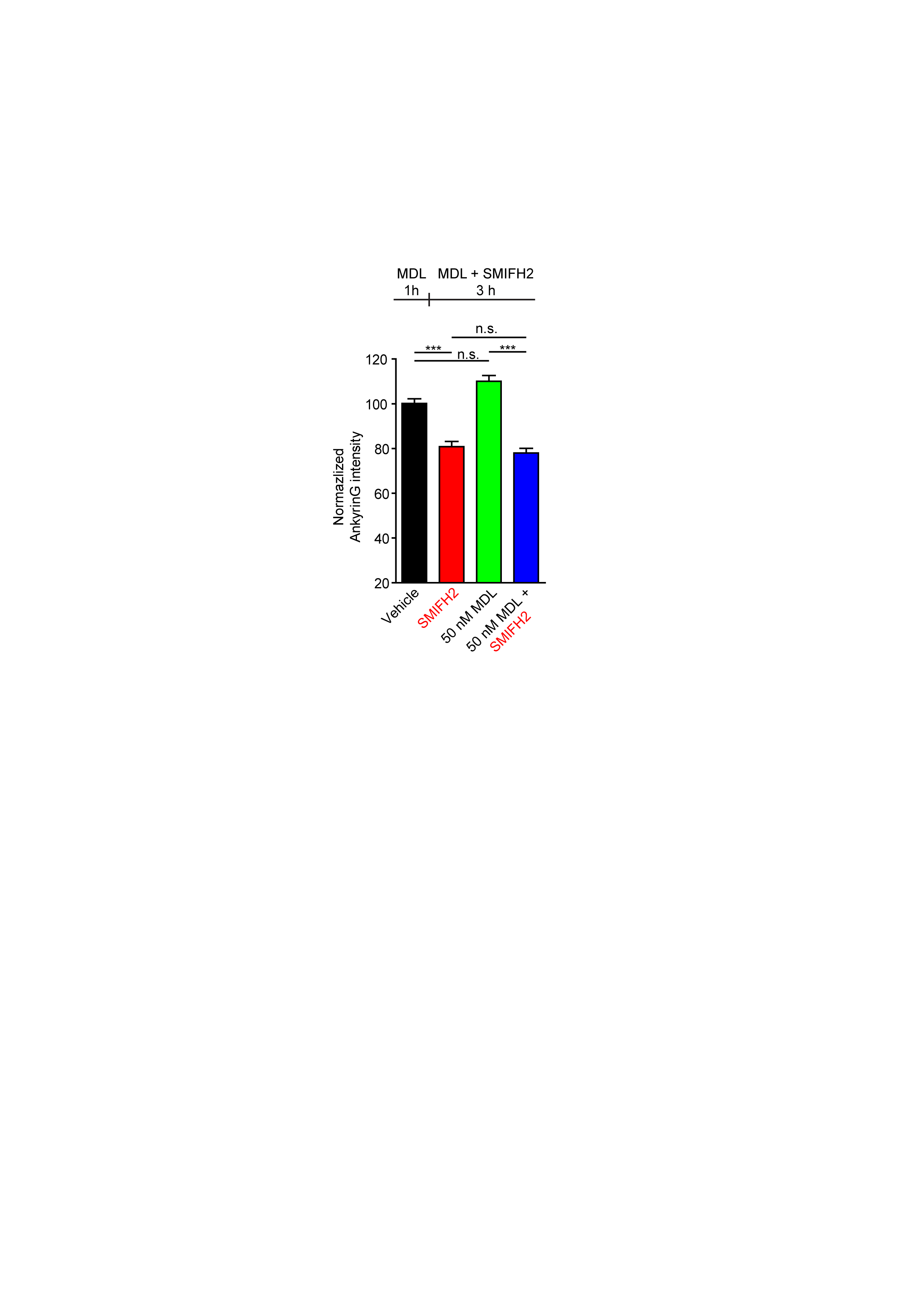

Supplement: Supplementary file 6 — High Resolution Image (TIF 162 kb) [file 12035_2021_2531_MOESM3_ESM.tif]

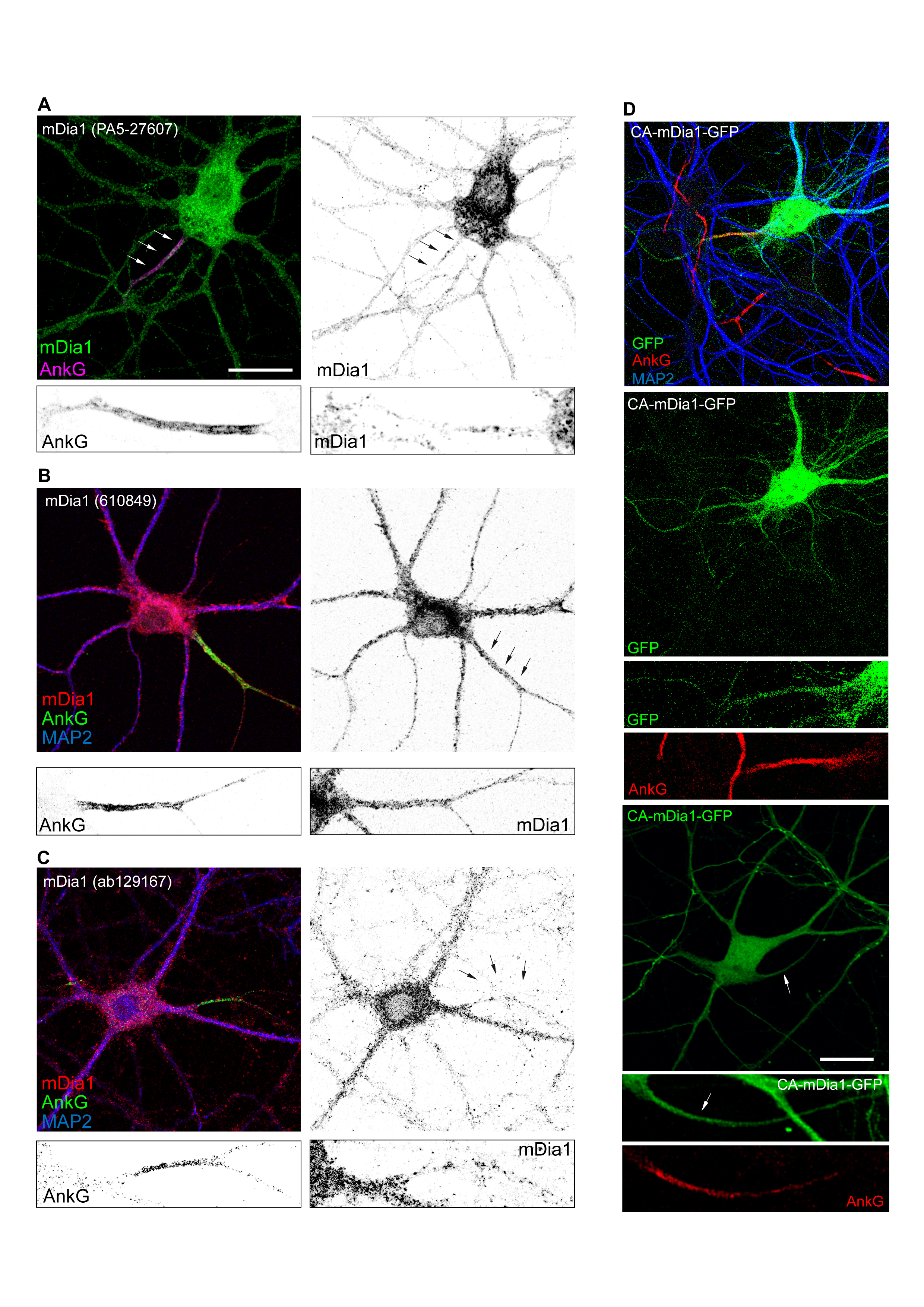

Supplement: Supplementary file 7 — mDia1 expression in hippocampal neurons (A-C) 14 DIV hippocampal neurons stained with 3 different mDia1 antibodies (PA5-27607, 610849, and the knockout validated antibody ab129167). AIS was identified by ankyrinG signal and dendrites by MAP2 staining. AIS region and mDia1 staining or ankyrinG stainings are magnified in bottom panels. Scale bar = 20 μm. (D) Two examples of 12 DIV neurons expressing GFP-CA-mDia1 (green) after transfection at 10 DIV. Neurons were stained with ankyrinG (red) and MAP2 antibodies (blue in upper neuron). (PNG 25497 kb) [file 12035_2021_2531_Fig11_ESM.png]

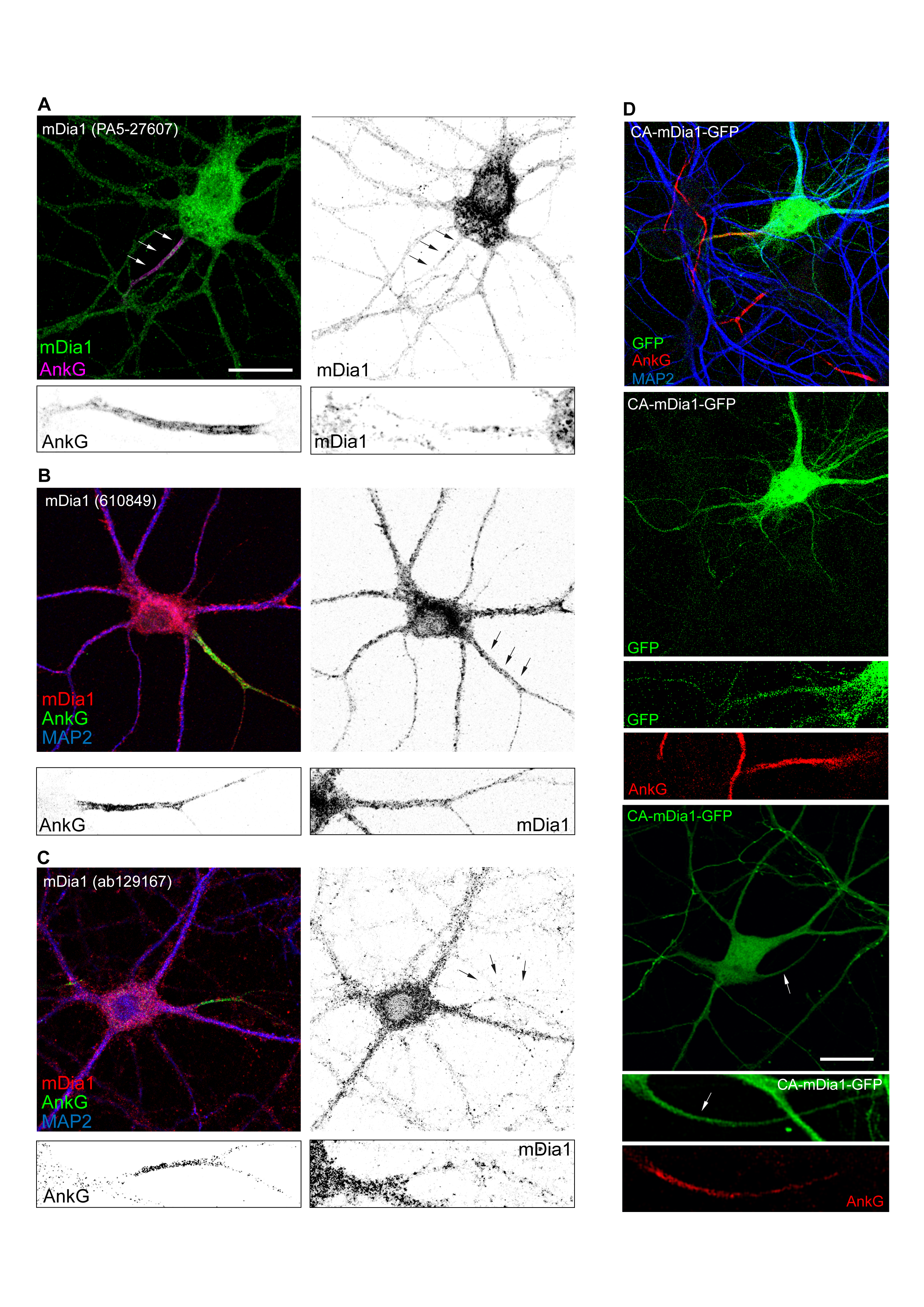

Supplement: Supplementary file 8 — High Resolution Image (TIF 6124 kb) [file 12035_2021_2531_MOESM4_ESM.tif]

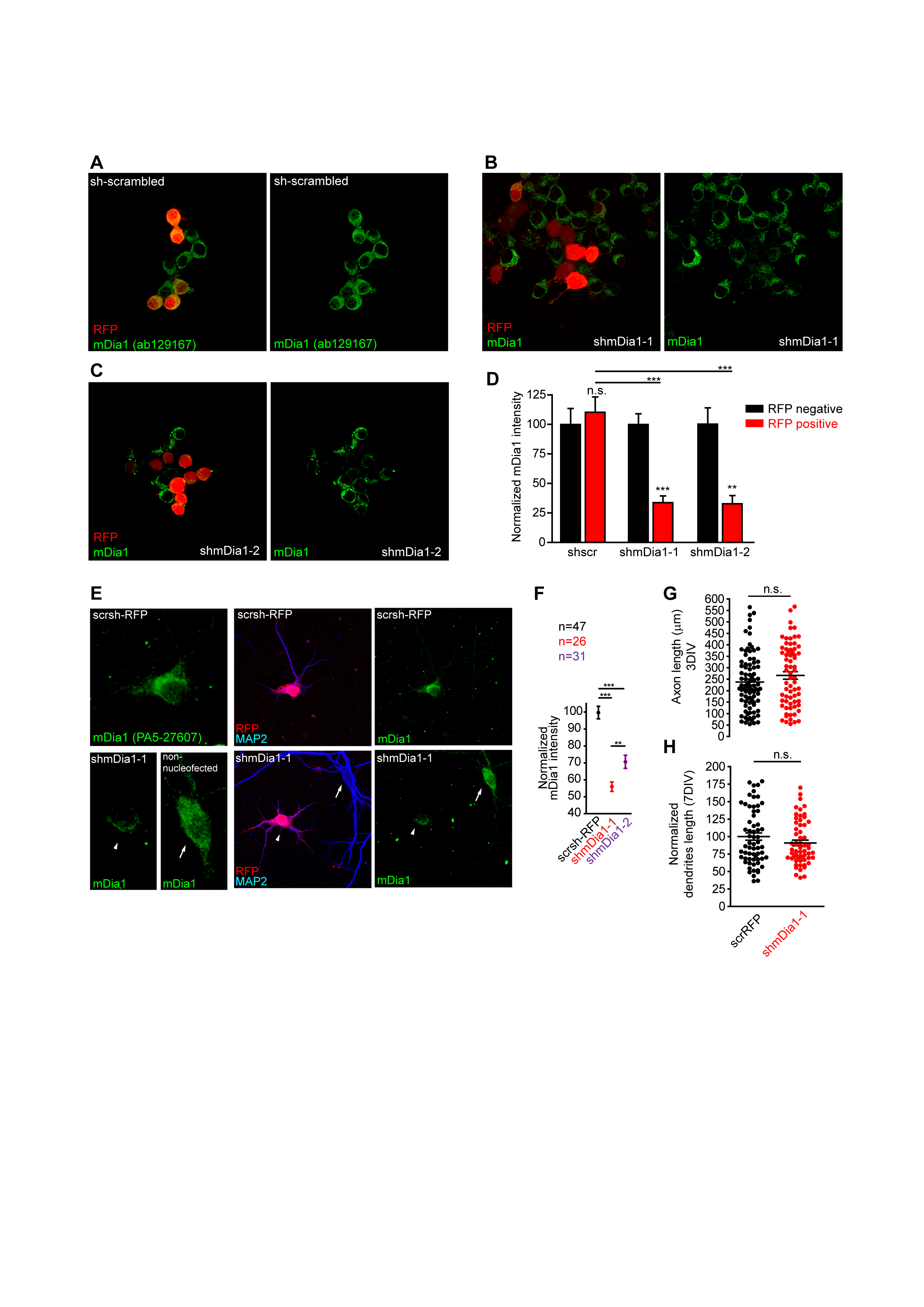

Supplement: Supplementary file 9 — (A-C) Neuro2a cells transfected with srambled (A), shmDia1-1 (B) or shmDia1-2 (C) interference shRNAs for 3 days. Transfected cells are identified by RFP signal (red) and mDia1 knockout validated antibody (ab129167, Abcam) signal is show in green. (D) Quantification of mDia1 signal in transfected (red bars) or non-transfected (black bars) Neuro2a cells (n=50 cells/experimental condition). Data are represented as the mean ± SEM. n.s., not significant, ** p < 0.01***, p < 0.0001, Mann-Whitney test. (E) 10 DIV hippocampal neurons nucleofected with scrambled interference RNA (scrsh-RFP) or mDia1 interference RNAs (shmDia1-1, shmDia1-2). Nucleofected neurons were identified based on RFP fluorescence (magenta). Neurons were stained with mDia1 (green) and MAP2 (blue) antibodies. Scale bar = 20 μm. (F) mDia1 fluorescence intensity was quantified in the soma of neurons nucleofected with scrsh-RFP, shmDia1-1 or shmDia1-2 plasmids. Inserts in D show magnifications of mDia1 staining in soma. **p < 0.01, ***p < 0.001, One-way analysis of variance, Tukey’s multiple comparison test. Data in graphs were acquired from three independent experiments and represented as the mean ± SEM. (G, H) Axonal and dendritic growth is not affected by mDia1 interference RNA. Hippocampal neurons were nucleofected before plating with shmDia1-1 interference RNA and kept for 3 DIV (G) to analyze axonal length using the tau1 axonal marker, or 7 DIV to analyze total dendritic length using the MAP2 somatodendritic marker (H). (PNG 25497 kb) [file 12035_2021_2531_Fig12_ESM.png]

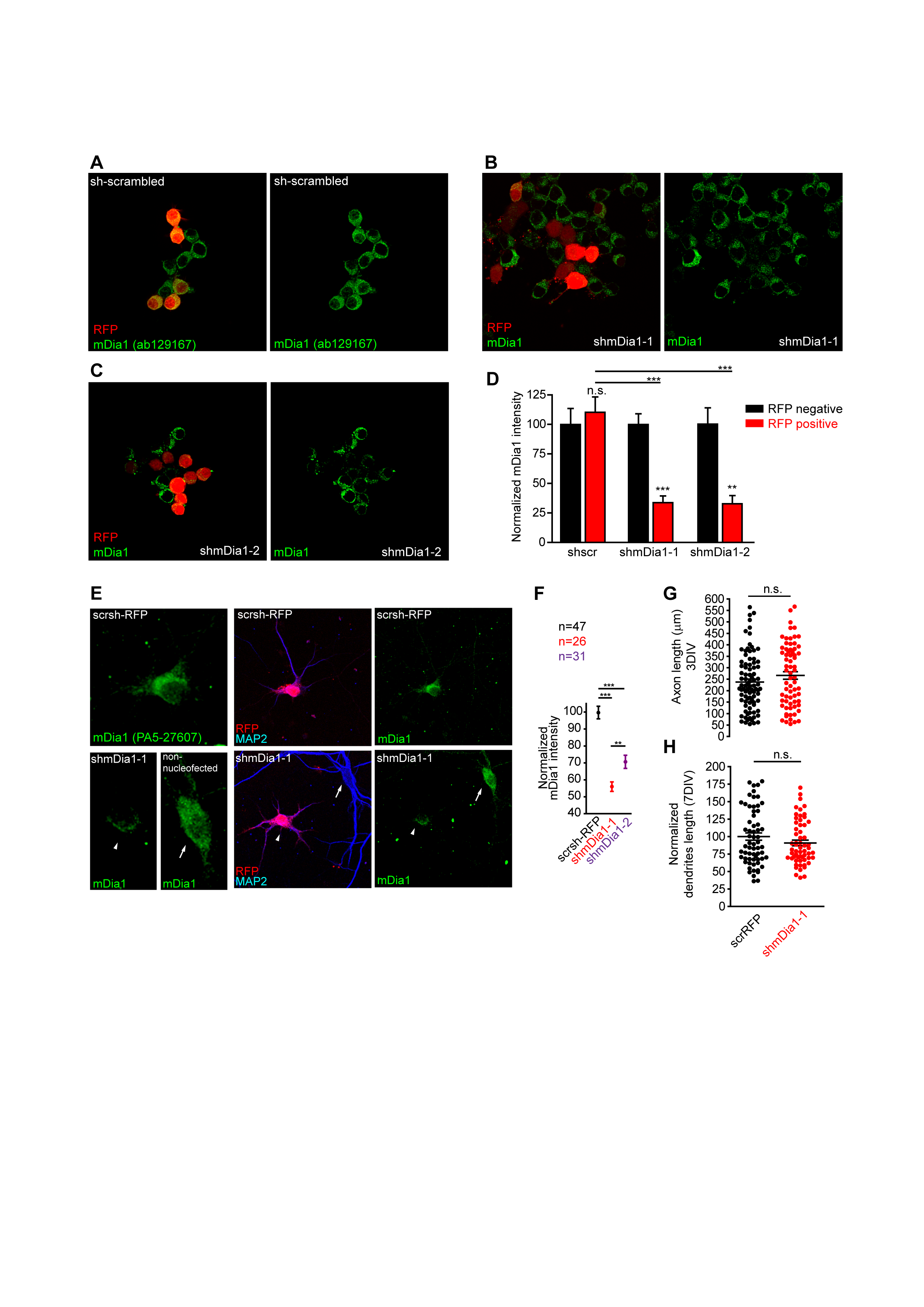

Supplement: Supplementary file 10 — High Resolution Image (TIF 1333 kb) [file 12035_2021_2531_MOESM5_ESM.tif]
